# The proteomic fingerprint in infants with single ventricle heart disease in the interstage period: evidence of chronic inflammation and widespread activation of biological networks

**DOI:** 10.3389/fped.2023.1308700

**Published:** 2023-12-08

**Authors:** Lindsay M. Thomson, Christopher A. Mancuso, Kelly R. Wolfe, Ludmila Khailova, Sierra Niemiec, Eiman Ali, Michael DiMaria, Max Mitchell, Mark Twite, Gareth Morgan, Benjamin S. Frank, Jesse A. Davidson

**Affiliations:** ^1^ Department of Pediatrics, University of Colorado Anschutz Medical Campus, Aurora, CO, United States; ^2^ Department of Biostatistics and Informatics, University of Colorado Anschutz Medical Campus, Aurora, CO, United States; ^3^ Department of Surgery, University of Colorado Anschutz Medical Campus, Aurora, CO, United States; ^4^ Department of Anesthesia, University of Colorado Anschutz Medical Campus, Aurora, CO, United States

**Keywords:** biomarkers, congenital heart disease, congenital heart defect, Glenn, hypoplastic left heart syndrome, inflammation, protein dysregulation, single ventricle palliation

## Abstract

**Introduction:**

Children with single ventricle heart disease (SVHD) experience significant morbidity across systems and time, with 70% of patients experiencing acute kidney injury, 33% neurodevelopmental impairment, 14% growth failure, and 5.5% of patients suffering necrotizing enterocolitis. Proteomics is a method to identify new biomarkers and mechanisms of injury in complex physiologic states.

**Methods:**

Infants with SVHD in the interstage period were compared to similar-age healthy controls. Serum samples were collected, stored at −80°C, and run on a panel of 1,500 proteins in single batch analysis (Somalogic Inc., CO). Partial Least Squares-Discriminant Analysis (PLS-DA) was used to compare the proteomic profile of cases and controls and t-tests to detect differences in individual proteins (FDR <0.05). Protein network analysis with functional enrichment was performed in STRING and Cytoscape.

**Results:**

PLS-DA readily discriminated between SVHD cases (*n* = 33) and controls (*n* = 24) based on their proteomic pattern alone (Accuracy = 0.96, *R*^2 ^= 0.97, *Q*^2 ^= 0.80). 568 proteins differed between groups (FDR <0.05). We identified 25 up-regulated functional clusters and 13 down-regulated. Active biological systems fell into six key groups: angiogenesis and cell proliferation/turnover, immune system activation and inflammation, altered metabolism, neural development, gastrointestinal system, and cardiac physiology and development.

**Conclusions:**

We report a clear differentiation in the circulating proteome of patients with SVHD and healthy controls with >500 circulating proteins distinguishing the groups. These proteomic data identify widespread protein dysregulation across multiple biologic systems with promising biological plausibility as drivers of SVHD morbidity.

## 1. Introduction

Single ventricle heart disease (SVHD) occurs in between 2 and 8 in 10,000 live births and is associated with some of the highest morbidity and mortality in patients born with congenital heart disease (CHD) (1). Most patients undergo a staged series of palliative surgical repairs, culminating in total cavopulmonary anastomosis. The first procedure, or stage 1, is performed shortly after birth, stage 2 (Glenn or hemi-Fontan) at 4–6 months of life, and a final stage 3 procedure (Fontan) between 2 and 5 years of age (2). The highest morbidity and mortality period occurs between stages 1 and 2, a particularly vulnerable time termed the “interstage period.” (3) During this time, the single ventricle pumps in parallel to the systemic and pulmonary circulations. The ratio of flow delivered to each circulation is dynamic and dependent on relative vascular resistances. This physiology results in chronic hypoxemia and leaves infants at risk of unbalanced circulation (excess or insufficient pulmonary blood flow), especially during times of illness, fever, dehydration, vagal events, or other effectors of pulmonary and systemic vascular resistance (3, 4).

Single ventricle physiology yields variable, and at times unstable, perfusion and oxygen delivery resulting in both acute and long-term sequelae across organ systems. Excess pulmonary blood flow (PBF) leads to adverse vascular remodeling and in the long-term causes pulmonary hypertension, hypoxemia, venous congestion, and congestive heart failure (5, 6). Conversely, insufficient PBF leads to severe hypoxemia (7). Dysregulated systemic blood flow results in differential organ-specific blood flow and, in combination with baseline hypoxemia, has whole-body downstream consequences. Examples include decreased cerebral blood flow, which correlates with lower IQ (8, 9), mesenteric ischemia resulting in necrotizing enterocolitis (NEC) (10), and chronic kidney disease from episodic low renal blood flow on a background of chronic hypoxia (11). While the multiorgan clinical sequelae of SVHD have been well-described, a gap exists in our understanding of the systemic derangement at the cellular and molecular levels. Moreover, we do not fully understand how individual molecular changes interact across larger biological systems and how those systems drive observed clinical sequelae.

Proteomic techniques may help address this gap. Proteomics allows for large-scale protein exploration to define the extent of individual protein dysregulation experienced by children with SVHD and the underlying biological networks. Our group has begun to utilize “omics” to explore the shifts in the global metabolome and proteome of patients with SVHD. Prior work from our group demonstrated a unique targeted cardiovascular proteomic profile in patients with SVHD compared to healthy controls (12). However, the breadth of circulating protein dysregulation experienced by infants with interstage SVHD has not been well defined. In this study, we performed an expanded analysis of proteins across a broad range of biological systems to characterize the circulating proteome in infants awaiting Stage 2 palliation compared to healthy controls. We hypothesized that the circulating proteome is widely affected by SVHD physiology with diffusely altered biological systems arising from the heart, kidneys, brain, gastrointestinal tract, and immune system.

## 2. Materials and methods

The Colorado Multiple Institutional Review Board approved this study. Written informed consent was obtained from the study subjects’ parents or legal guardians in all cases*.* This study is a pre-specified aim of a larger parent study examining longitudinal circulating biomarkers of pulmonary vascular development in SVHD patients.

### 2.1. Subjects

We prospectively enrolled patients at our center with SVHD in the interstage period prior to their Stage 2 surgical palliation. We defined Stage 2 palliation to include any form of superior cavopulmonary anastomosis (Glenn or Hemi-Fontan operations) regardless of having had a Stage 1 palliation. In addition, we recruited similar-aged control subjects from our institution's broader non-cardiac surgical schedule, who were aged 3 to 12 months and undergoing anesthesia for elective, non-cardiac procedures. We excluded control subjects with known or suspected cardiac, pulmonary, infectious, or genetic abnormalities. Complete details regarding inclusion and exclusion criteria for the parent cohort have previously been published (12).

### 2.2. Sample collection and processing

All samples were collected under general anesthesia. When possible, systemic venous samples from SVHD subjects were obtained at the time of pre-stage 2 cardiac catheterization. Otherwise, a systemic venous sample was collected in the CVOR prior to the first incision. For control subjects, we obtained systemic venous samples after induction of anesthesia at the time of IV placement. All whole blood samples were processed for serum at the time of collection and then aliquoted into 200 μl samples and frozen at −80°C until analysis.

### 2.3. Protein quantification assays

We analyzed samples in a single batch using a DNA-based aptamer assay (SomaLogic, Boulder, CO) with a custom panel of 1,500 proteins selected to represent markers of organ function from a wide variety of systems (Supplementary Table S1). For each protein target, chemically labeled nucleotide-antibody pairs were bound to specific epitopes on the protein surface. Complementary nucleotide sequences gave rise to DNA reporter sequences, which were then quantified using real-time PCR (13). The assay measured a dynamic range of absolute protein quantities across 10 logs with a sensitivity down to 125 fM (14). Log-scaled normalized protein expression values were adjusted by a negative control sample. Higher expression values correspond to higher protein levels but are not an absolute quantification of protein concentrations. Given that chronic dysregulation at the organ level may be associated with only modest alterations in the circulating proteome, we elected to err on the side of inclusivity and not impose a low-fold change filter.

### 2.4. Identification of significant proteins

We used Metaboanalyst 5.0 (www.metaboanalyst.ca, RRID:SCR_015539) (15) to find differences in the proteome between cases and controls. Protein concentrations were log-transformed and auto-scaled (mean-centered and divided by the square root of the standard deviation of each variable) for normalization. We then analyzed the normalized data for global proteome differences using Partial Least Squares-Discriminant Analysis (PLS-DA) followed by model cross-validation (R^2^, Q^2^). Variable Importance in Projection (VIP) multivariate regression identified the top 35 proteins distinguishing cases and controls. For cases alone, secondary analyses to assess the association between key covariates and the global proteome were done using Principal Component Analysis (PCA). Covariates tested included sex, ventricular morphology, and history of surgical Stage 1 palliation. As cases and controls were not perfectly matched for age or weight, we sought to rule out a significant proteomic signal related to variation of age and weight. To do so, we performed simple linear regressions in controls, separately comparing each protein with age and weight and correcting for multiple comparisons using the Benjamini-Hochberg method (i.e., False Discovery Rate, FDR) (16). To identify individual proteins that differed between cases and controls, we then performed t-tests on the entire panel to identify all proteins that differed significantly between cases and controls with FDR multiple comparisons correction. We defined the findings to be statistically significant at an FDR <0.05. All further protein analysis was performed on the significantly different proteins alone.

### 2.5. Protein-protein interaction analysis

Due to the incomplete measurement of the proteome inherent even to large targeted proteomics assays such as ours, we used a network-based method, GenePlexus (17, 18), to infer additional untested proteins strongly related (>99% probability) to the original significant protein list. We then imported this expanded list of statistically significant proteins into STRING (string-db.org, RRID:SCR_005223) (19) to create protein-protein interaction (PPI) networks. Interactions were determined by a combination of evidence sources, including experimental data, co-expression, co-occurrence, gene fusion, neighborhood interactions, and literature text mining. As this work was exploratory in nature, we constrained interactions to “medium confidence” interactions (i.e., setting 0.4 as the minimum required interaction score). We created two primary PPI networks: one for up-regulated and one for down-regulated proteins. To understand functional subnetworks within each larger PPI network, we clustered up-regulated and down-regulated differentially expressed proteins (DEPs) using Markov Clustering (MCL) with an inflation parameter of 3.0. We further clustered large (≥100 proteins) clusters using an inflation parameter of 5.0 to drive cluster granularity, such that no single sub-cluster contained greater than 99 proteins (20). All proteins are referred to by their gene name according to the HUGO Gene Nomenclature Committee naming system (RRID:SCR_012800).

### 2.6. Functional enrichment analysis: classification of biological networks and systems

Functional enrichment analysis was performed using STRING, specifically looking at the enrichment of Gene Ontology (GO) biological processes. First, we performed GO analysis on the broader up and down-regulated networks as well as each cluster and sub-cluster (21, 22). We then grouped individual GO terms identified at the cluster level into biologically similar groups to better understand the broader biological networks and systems. For grouped GO terms, the lowest *p*-value in the group is reported. Finally, we imported clusters into Cytoscape (23) (RRID:SCR_003032) to analyze node degrees, a numerical reflection of the connectivity of each protein (node), and edges (total number of connections in the cluster). Cluster visualization was performed in Cytoscape.

## 3. Results

### 3.1. Study population

We enrolled 33 patients with SVHD prior to Stage 2 palliation and 24 controls. All patients were included in the analysis and pre-operative systemic vein samples were available for all subjects. SVHD subjects had various underlying diagnoses, the most common being hypoplastic left heart syndrome (HLHS). Of the 33 SVHD patients, 27 were palliated to a single right ventricle, and the remaining six were palliated to a single left ventricle (Table 1). Twenty-two of 33 single ventricle patients had previously undergone a Norwood operation. The remaining 11 patients had either not undergone Stage 1 surgical repair or had a more limited initial palliation (e.g., systemic to pulmonary artery shunting alone or pulmonary artery banding). PCA demonstrated no significant differentiation of the circulating proteome by sex, ventricular morphology, or completion of Stage 1 palliation (Supplementary Figure S1). The most common surgical indications in controls were urogenital malformations or elective urogenital procedures. Owing to the elective nature of these procedures, controls were, on average older and heavier, differences which met statistical significance. The average age at the time of sample collection was 3.41 (±1.19) months in cases and 8.42 (±2.20) months in controls. The average weight was 5.78 (±0.66) kg in cases and 8.07 (±0.89) kg in controls. Simple linear regression analysis identified no statistically significant relationship in any protein with age or weight (FDR <0.05).

**Table 1:** Demographics.

	Control (*n* = 24)	Case (*n* = 33)	*p* value
Weight, kg			
Mean ± SD	8.07 ± 0.89	5.78 ± 0.66	0.0001
Median [min, max]	7.80 [6.58, 10.38]	5.7 [4.5, 7.2]	
Age, m			
Mean ± SD	8.42 ± 2.20	3.41 ± 1.19	0.0001
Median [min, max]	[5.12, 11.66]	3.41 [0.85, 5.54]	
Sex			
Female	8 (33.33)	12 (36.4)	
Male	14 (58.33)	21 (63.6)	
Ventricle Morphology			
LV		6 (18.2)	
		27 (81.8)	
Stage 1 operation			
Norwood		21 (63.6)	
Other		5 (15.2)	
PA band		1 (3.0)	

Values are *n* (%) unless otherwise indicated. LV, left ventricle; RV, right ventricle; PA, pulmonary artery.

### 3.2. Comparison of case and control proteomic patterns and differential protein expression

**Figure 1:**
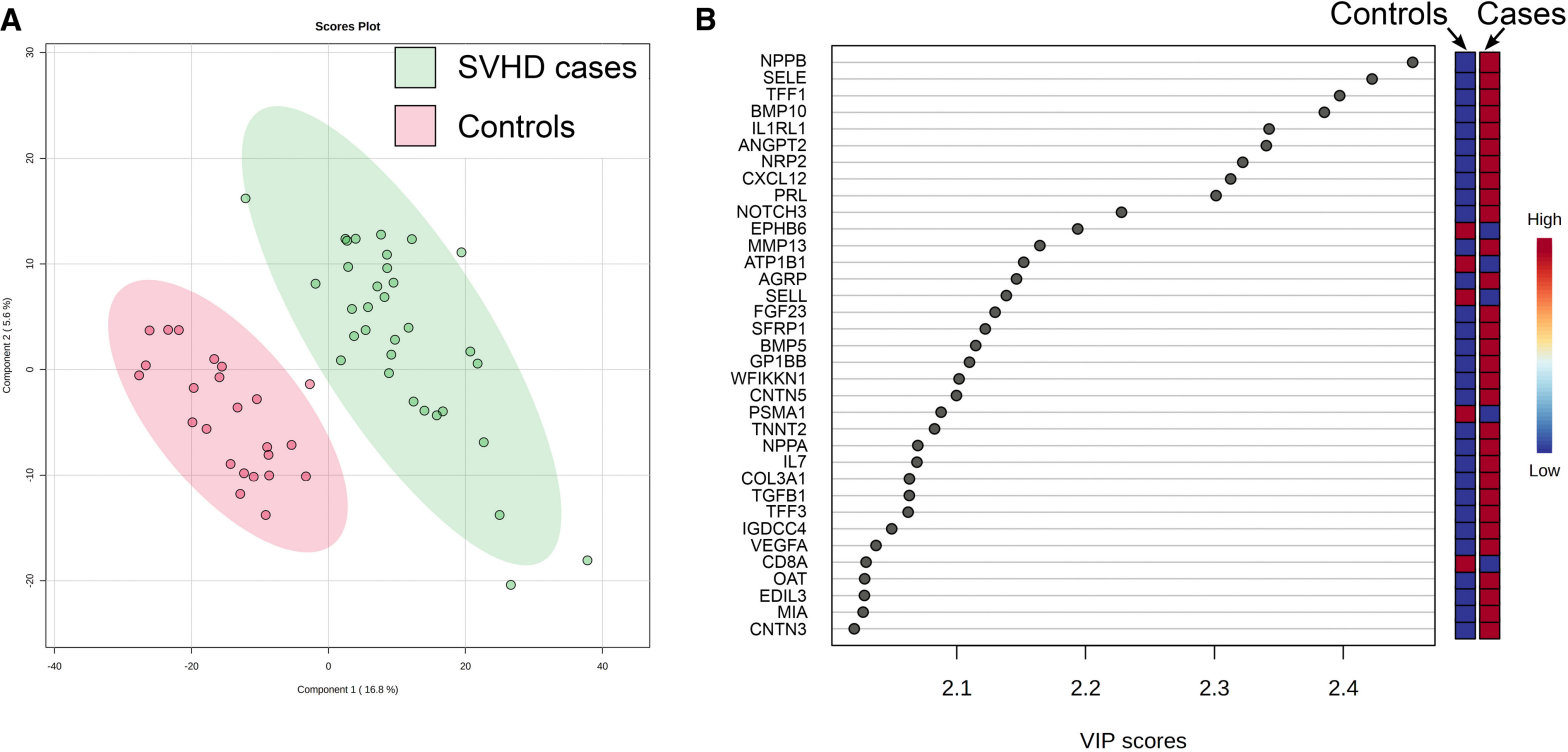
(**A**) Partial least squares-determinant analysis (PLS-DA) readily discriminated between SVHD cases (*n* = 33) and controls (*n* = 24) based on their proteomic pattern alone. Each circle represents the distilled proteome of one subject. (**B)** Variable Importance in Projection (VIP) analysis shows the 35 proteins with the highest effect in distinguishing the proteomes of SVHD cases and controls. Relative up and down-regulation of each protein (high vs. low) demonstrates an overall trend toward up-regulation in SVHD cases.

PLS-DA demonstrated clear discrimination between SVHD cases and controls based on their proteomic patterns alone (Accuracy = 0.96, R^2 ^= 0.97, Q^2 ^= 0.80) (Figure 1A). VIP identified the 35 proteins contributing most to the predictive power of the PLS-DA model and thus on proteome differentiation (Figure 1B). Thirty of the top 35 proteins were up-regulated in SVHD patients, functioning across multiple biological systems. The biological processes most represented include angiogenesis, immune system regulation, and neuronal morphogenesis. The protein with the single highest effect in distinguishing cases and controls was NPPB, coding for B-type Natriuretic Peptide (BNP). T-tests showed 568 proteins differed significantly between groups at FDR <0.05. In total, 366 proteins were up-regulated, and 202 were down-regulated, demonstrating an overall trend toward protein up-regulation in SVHD patients.

### 3.3. Protein-protein interaction analysis and functional enrichment

First, the original list of significant proteins was expanded using the network-based computational method, GenePlexus. GenePlexus identified 19 additional proteins that had a >99% probability of being associated with the original set. We imported these 587 DEPs into STRING and generated a network with 586 nodes and 7,047 edges. The PPI enrichment for the network, a value estimating the number of observed vs. expected protein interactions for a specific network size, was found to be highly statistically significant (*p* < 1 × 10^−16^).

**Figure 2:**
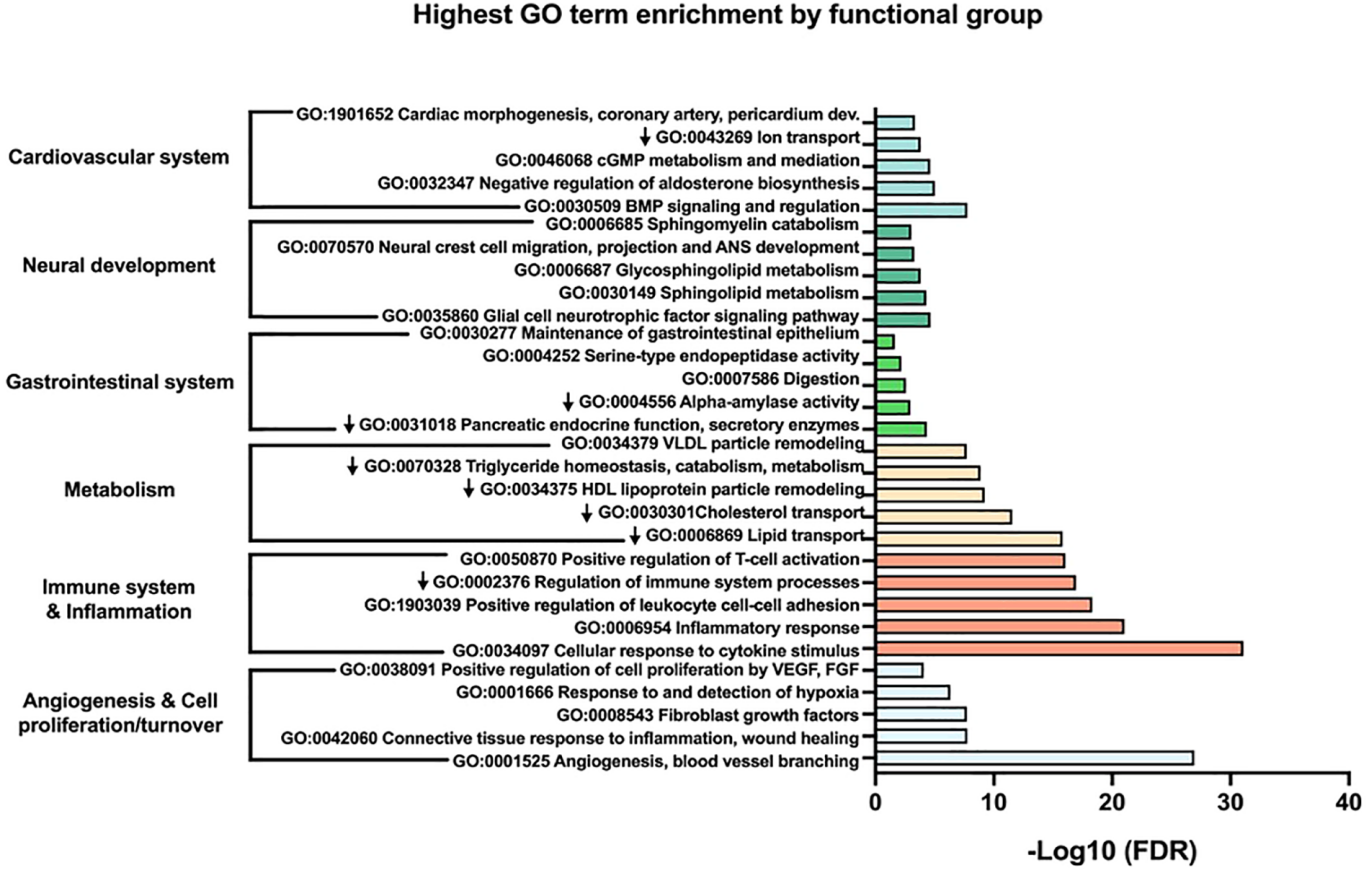
The top five most significant gene ontology (GO) terms within each larger functional group are shown. Down arrows signify GO terms from down-regulated clusters.

Next, we performed a more in-depth analysis of the up and down-regulated protein lists separately. Running GenePlexus on each of these lists identified 20 additional proteins to the up-regulated protein set, for a total of 386 up-regulated proteins. No additional proteins were identified in the down-regulated group. To discover the active biological systems in our cases, we performed MCL on the up and down-regulated networks separately. MCL identified 25 major (≥3 nodes) clusters in up-regulated proteins (Table 2) and 13 major clusters in down-regulated proteins (Table 3). We grouped enriched GO terms across six broad categories (Figure 2). Most unified GO groups consisted of multiple clusters. A list of unifying GO terms assigned to each cluster can be found in Supplementary Tables S2 and S3.

**Table 2:** Significant Up-regulated clusters and proteins.

**Cluster 1a**	IGFBP7	ADAMTS4	IL1B	ING1	**Cluster 13**	**Unclustered or cluster size ≤2**	GADD45	PCDH17
ADA	KL	ADAMTS5	IL1RL1	PON1	CASP2		GDF15	PEAR1
ADAM12	LIF	MMP16	IL24	SERPINA11	CHEK2	ACY1	GGH	PI3
AFP	LTBP4	**Cluster 1c**	IL3	TFPI	STK4	ADAM9	GH2	PPCDC
ANGPT2	LUM	FGF9	IL6	**Cluster 5**	**Cluster 14**	ADAMTS1	GOLM2	PPIB
ANGPT4	MCAM	FGF6	IL6ST	ACO1	CFC1	AGXT	GP1BB	PRSS2
ANGPTL2	MIA	FGF5	IL7	ALDH3A1	LEFTY2	AIF1l	GZMH	PSAP
BSG	MMP13	FGF20	IL7R	MPI	NODAL	ALCAM	HAPLN1	PTEN
CCDC80	MMP17	FGF17	LEP	PC	**Cluster 15**	ANGPTL1	HMGCL	PTN
CD248	MMP2	**Cluster 1d**	MNDA	PCK2	APP	APOH	HMGCS1	PYY
CDH5	MMP7	ADAMTS13	NPPA	RBKS	CNTN1	ASL	HSPG2	RAB11FIP3
CILP	NID1	SPON2	NPPB	TALDO1	SNCG	ATP6V1F	IGDCC4	RBP5
COL15A1	NOTCH1	THBS2	PLA2G10	**Cluster 6**	**Cluster 16**	B4GALT1	IGF2R	RP2
COL18A1	NOTCH3	**Cluster 2**	PLAT	GFRA1	ENPP7	CA11	IGFBP4	RRM2
COL3A1	NRP2	AGRP	PRL	GFRA3	GM2A	CA12	IGSF3	S100A5
COL4A2	NTF3	ANGPTL4	PTGS2	NTRK3	SMPD1	CA6	INHBA	SARG
COL9A1	NTRK2	CCL14	PTX3	PSPN	**Cluster 17**	CCN2	ITGA5	SCARB2
CTGF	OGN	CCL15	RARRES2	RET	ASPN	CD109	ITGB5	SFRP1
CTHRC1	PAK4	CCL16	REN	**Cluster 7**	EEF1D	CD2	JAM2	SGSH
DCN	PCOLCE	CCL2	S100A12	NPPC	FNDC1	CDHR1	KCNE5	SLIT3
DLK1	PDGFRA	CCL20	SELE	PDE2A	**Cluster 18**	CDKN1A	KEL	SMAD3
DLL4	PDGFRB	CCL24	SIRPA	PDE3A	CD22	CEP43	KYNU	SOD1
DPT	PECAM1	CCL27	STAT3	PDE5A	PHOSPHO1	CGA	LAYN	SOD2
DUSP3	PGF	CD38	STAT5B	PDE7A	SIGLEC15	CGREF1	LGALS2	TACSTD2
EFNA1	PLAU	CD4	TLR2	**Cluster 8**	**Cluster 19**	CHGA	LGALS3BP	TFPI2
EFNA4	PLAUR	CD40LG	TNFSF10	PRSS1	ACOX1	CHL1	LRCH4	TGFBI
EGFR	POSTN	CD47	TNNI3	PRSS3P2	FABP1	CLPS	LRRN1	TINAGL1
ENG	PTK7	CD5	TSLP	SPINK1	SCP2	CLUL1	LY6D	TMEM132A
EPHA1	PTPRS	CD6	TXLNA	TMPRSS15	**Cluster 20**	CNTN3	MAT1A	TNC
EPHB4	RSPO1	CD70	**Cluster 3**	**Cluster 9**	GKN1	CNTN4	MATN3	TNFRSF10A
FAP	S100A4	CLEC10A	BAMBI	ABL1	TFF1	CNTN5	MDK	TNFRSF6B
FBN1	SPP1	CLEC11A	BMP10	BOC	TFF3	CPE	MEP1A	TNNT2
FGF18	TEK	CRP	BMP2	ROBO2	**Cluster 21**	CPQ	MET	TPP1
FGF3	TGFB1	CXCL10	BMP4	SLIT2	AKR1C4	CST3	METAP1	UGDH
FGF4	TGFB3	CXCL12	BMP5	**Cluster 10**	FABP6	CTSB	MFGE8	VIP
FGFR1OP	THBS4	CXCL16	BMP6	FUT3	RBP2	DAG1	MYDGF	VSIG10l
FLRT2	THY1	CXCL8	FSTL3	GALNT3	**Cluster 22**	DCTPP1	NCAM1	VWC2
FLT1	TIMP1	CXCR4	PAEP	GCNT1	PIGR	EDIL3	NCAM2	VWF
FLT4	TIMP2	DDR1	SMAD1	ST6GAL1	TF	EDN2	NDUFA2	WFDC2
FMOD	TNXB	EDN1	SOST	**Cluster 11**	TFRC	ENPEP	NFYA	
FN1	VEGFA	EPO	WFIKKN1	HMOX2	**Cluster 23**	ERBB2	None	
FSTL1	VEGFC	HAVCR2	**Cluster 4**	PON2	LRP1	FABP4	NPL	
GPNMB	VIM	ICAM1	ANGPTL3	POR	LRPAP1	FGF12	NRP1	
HGF	**Cluster 1b**	ICAM3	APOA2	**Cluster 12**	**Cluster 25**	FGF23	NTF4	
HYAL1	ADAMTS12	IL11	APOB	ARHGAP1	CDSN	FGFBP1	OAT	
HYAL2	ADAMTS2	IL18R1	APOC1	PRKCQ	DSC2	FKBP7	OXT	
IGF1R	ADAMTS3	IL19	DBI	RHOC	KLK10	FRZB	PAM	

All significantly up-regulated proteins, organized by cluster. Unclustered proteins and proteins associated with cluster sizes of 2 or fewer proteins are included in the last three columns. Proteins highlighted in blue were added via GenePlexus.

**Table 3:** Significant down-regulated clusters and proteins.

**Cluster 1**	KRT19	ECE1	**Unclustered or cluster size ≤2**	IGFBP6
BIRC3	LAMA4	EDN3		IRAG2
BMP1	LCP1	GAL	ADGRE5	KITLG
BMP15	NAMPT	LGALS1	ADGRG2	KYAT1
BMP8B	NCR1	LGALS3	AHSP	LTA
BMPR1A	NFATC1	**Cluster 5**	ANGPTL1	LTBR
BMPR1B	OSM	BCAN	APOL3	MAMDC2
CCL25	OSMR	CRTAC1	ART3	MANSC4
CD14	PDCD1	OMG	ATP1B1	MYOC
CD209	PPARG	SEMA4D	ATP1B3	NDUFB11
CD34	PTPN1	TNR	BACH1	NECTIN2
CD46	PVRL2	**Cluster 6**	C1GALT1C1	NPTX1
CD48	SELL	AGR2	C1QTNF1	NPTXR
CD7	SIRT1	CD59	CBLIF	NUCB2
CD80	SIRT2	CD97	CBS	NUDT10
CD8A	SIRT3	LYPD3	CD300C	PDE9A
CDH1	SLAMF1	**Cluster 7**	CDA	PMVK
CDH3	SYN3	ENO3	CDHR5	PSMA1
CDH6	THPO	GNPDA1	CES1	PSME1
CLDN1	TLR5	HK1	CETN2	PYDC1
CR2	TNF	PFKM	CPM	RASSF2
CRTAM	**Cluster 2**	**Cluster 8**	CPN2	RELT
CSF1	ACTN4	AIFM1	CRYZL1	RGS8
CSF3	AQP4	CD99L2	CSDE1	SBSN
DSG2	DPP6	HSD11B1	CSH1	SHD
ERBB3	FABP3	L3HYPDH	CSPG4	SPINK8
FCER2	KCNIP4	**Cluster 9**	DNPH1	STAB2
FEN1	MYL12B	RLN3	DSG3	TMPRSS5
FLT3	NEFL	GIP	DSG4	TOR1AIP1
FOXO3	PKD2	FSHB	EFEMP1	TPH1
FURIN	SCN4B	**Cluster 10**	ENOX2	TPPP3
GIF	TNNT3	CBSL	ENPP6	TPSAB1
HIF1A	TPM3	MAT2A	ENTPD6	TREML2
ICAM2	**Cluster 3**	MTAP	EPHB6	TRIAP1
IFNG	APOA1	**Cluster 11**	ERVV-1	
IL12B	APOA4	RPS10	FAM172A	
IL18BP	APOA5	SIL1	FAM3B	
IL18RAP	APOC2	TPT1	FCN2	
IL2RB	APOD	**Cluster 12**	GALNT2	
IL32	APOL1	AADAT	GBP6	
IL4	APOM	CCBL1	GPC1	
IL5RA	CPTP	GGCT	GSTA3	
ITGB2	LCN1	**Cluster 13**	GSTP1	
ITM2A	MSR1	AMY2A	HBZ	
JUN	SCARB1	AMY2B	HOXD4	
KLK3	**Cluster 4**	CPA2	ICA1	
KRT17	CLC		IDI2	

All significantly down-regulated proteins, organized by cluster. Unclustered proteins and proteins associated with cluster sizes of 2 or fewer proteins are included in the last two columns.

### 3.4. Angiogenesis and cell proliferation/turnover

**Figure 3:**
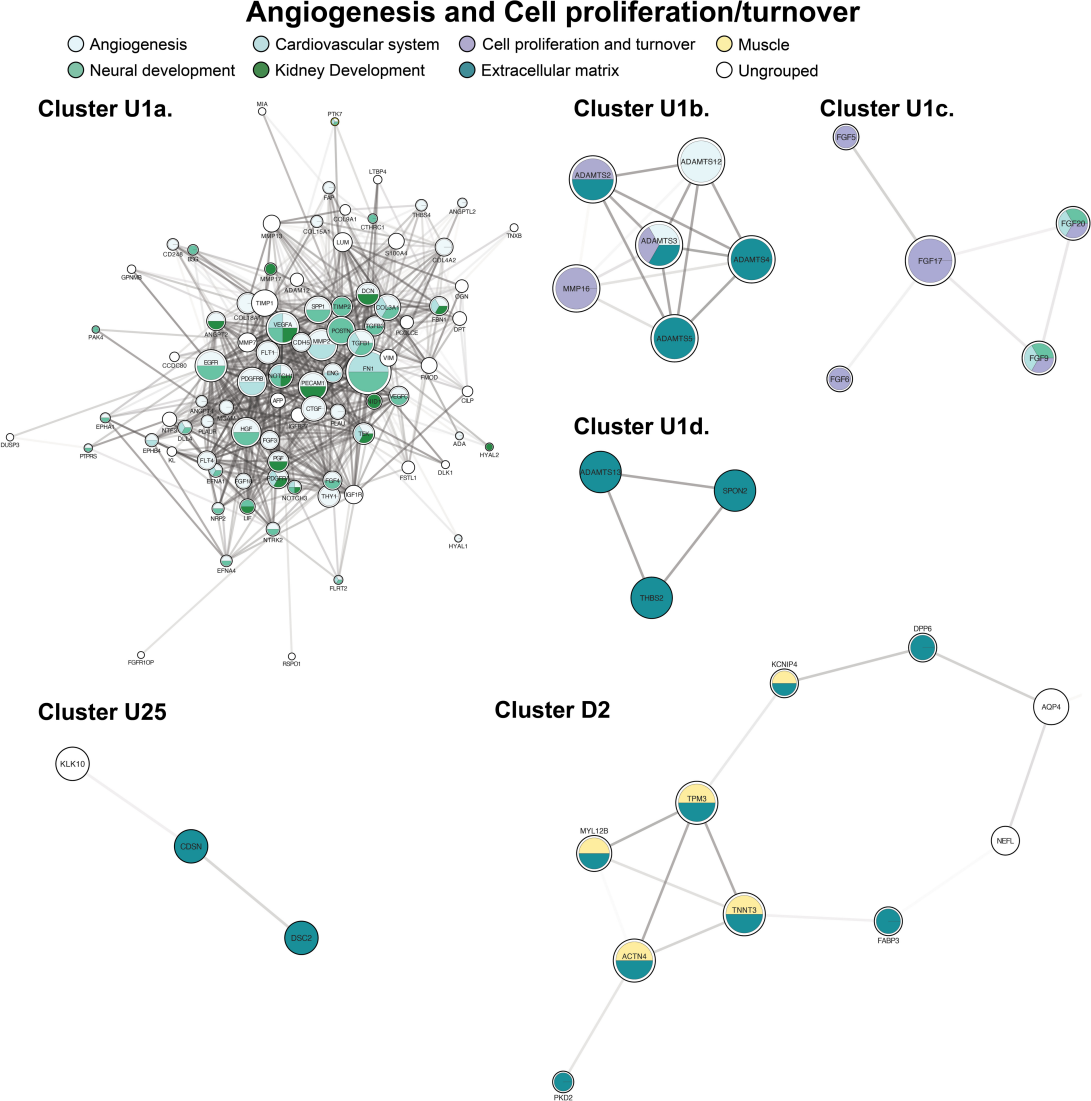
Clusters of angiogenesis and cell proliferation and turnover. Clusters with a “U” designation were up-regulated and clusters with a “D” designation were down-regulated. Figure protein (node) size is reflective of the degree of connectivity of the protein. Transparency of connecting lines (edges) is reflective of the “interaction score”, or the relative confidence in interaction by STRING.

The largest and most highly connected protein cluster in our network, up-regulated cluster 1, centered on angiogenesis and cell proliferation/turnover (Figure 3). Cluster 1 had 112 proteins by initial clustering. Using an inflation factor of 5, we generated four sub-clusters, with cluster U1a remaining large at 89 proteins. This large sub-cluster comprised angiogenic factors (Figure 3, cluster U1a). There are no down-regulated clusters mediating angiogenesis. The remaining sub-clusters were centered on cell proliferation and extracellular matrix (ECM) changes (Figure 3, clusters U1b-d). Two additional clusters also contained ECM proteins (Figure 3, clusters U25, D2).

### 3.5. Immune system activation and inflammation

**Figure 4:**
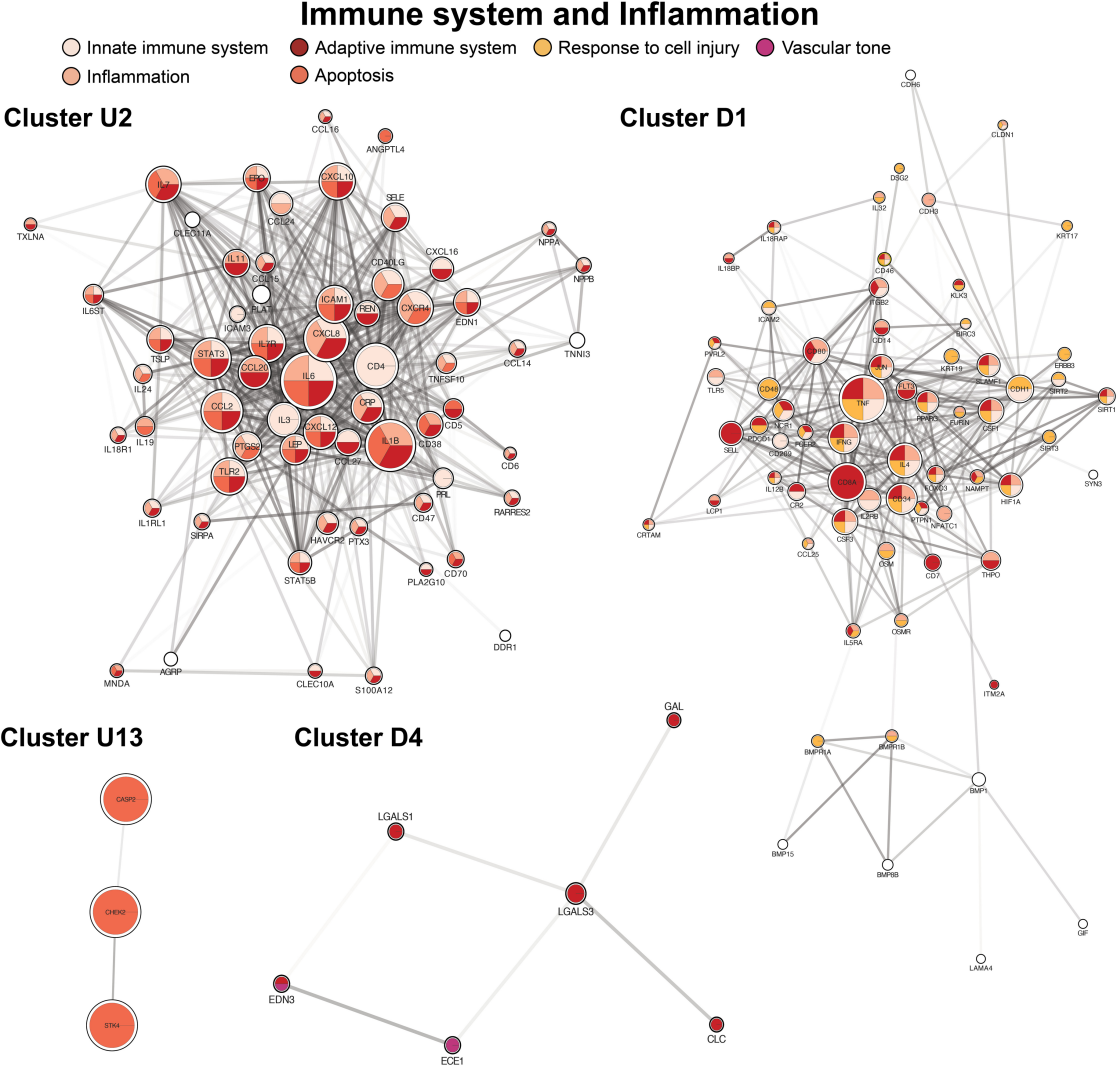
Clusters of immune system regulation, inflammation, response to cell injury (antioxidants), and apoptosis. Cluster formatting is described in Figure 3.

The second largest cluster, cluster U2, contained 69 proteins and focused on immune system regulation with co-expression of inflammatory cascades (Figure 4, cluster U2). A predominant group of proteins acted as activators of the adaptive immune system. Two down-regulated clusters were also highly enriched by adaptive and innate immune system mediators (Figure 4, clusters D1, D4). Apoptotic proteins were included within several clusters (principally Figure 4, clusters U1, U13). In contrast, antioxidants were among the proteins expressed in the principal down-regulated immune cluster (Figure 4, cluster D1).

### 3.6. Altered metabolism

**Figure 5:**
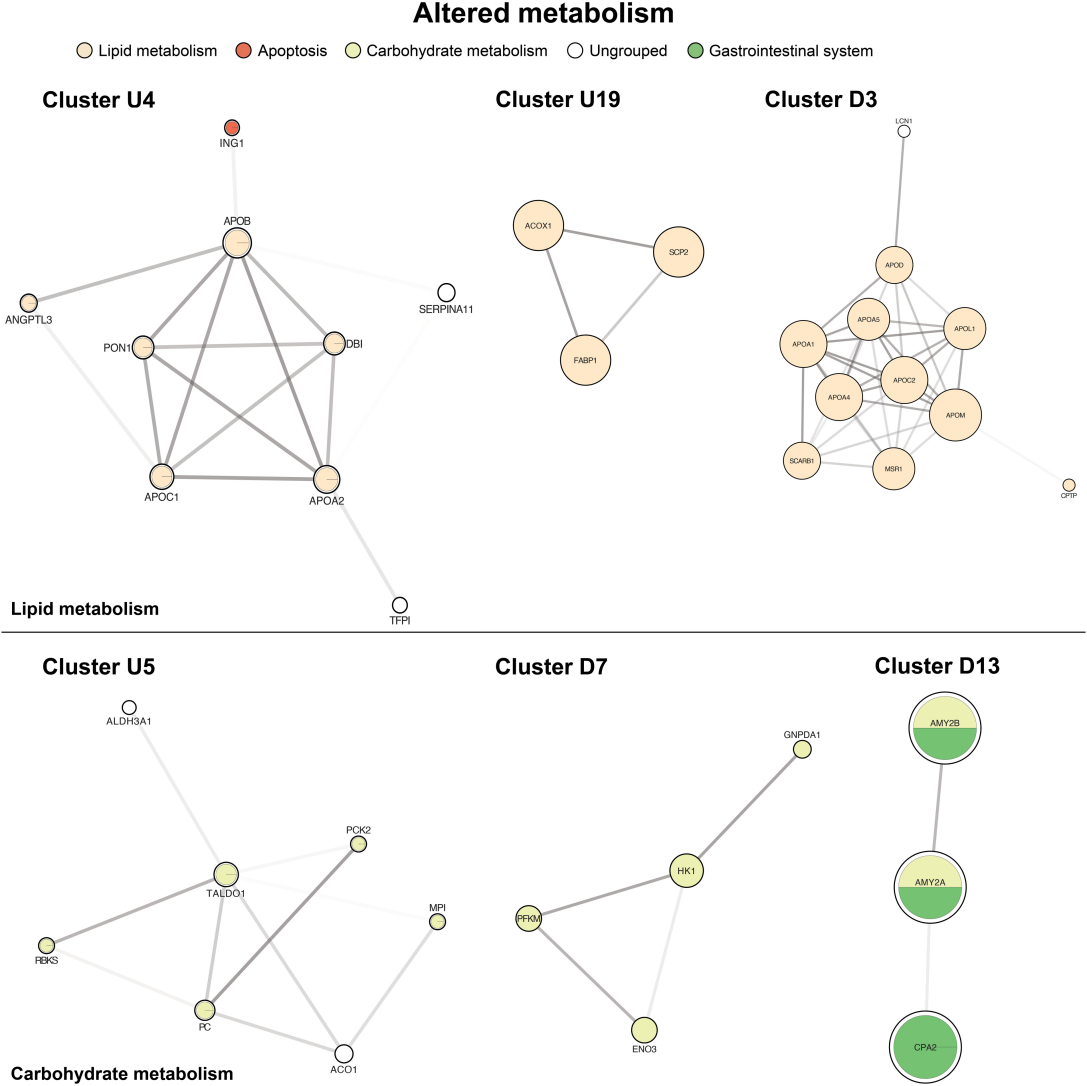
Of altered metabolism. The top panel is centered on lipid metabolism (U4, U19, D3). The bottom panel shows carbohydrate metabolism and digestion (U5, D7, D13). Cluster formatting is described in Figure 3.

Key metabolic processes appeared in both the up-regulated and down-regulated protein networks. The primary groups governed lipid and carbohydrate biosynthesis, processing, and transport (Figure 5, clusters U4, U19, D3). The second most represented metabolism pathways centered on carbohydrate metabolism and digestion with both up and down-regulators (Figure 5, clusters U5, D7, D13). We also identified smaller, distinct down-regulated clusters for deranged amino acids (e.g., tryptophan) and nicotinate and nicotinamide metabolism (Supplementary Figure S2).

### 3.7. Gastrointestinal system

**Figure 6:**
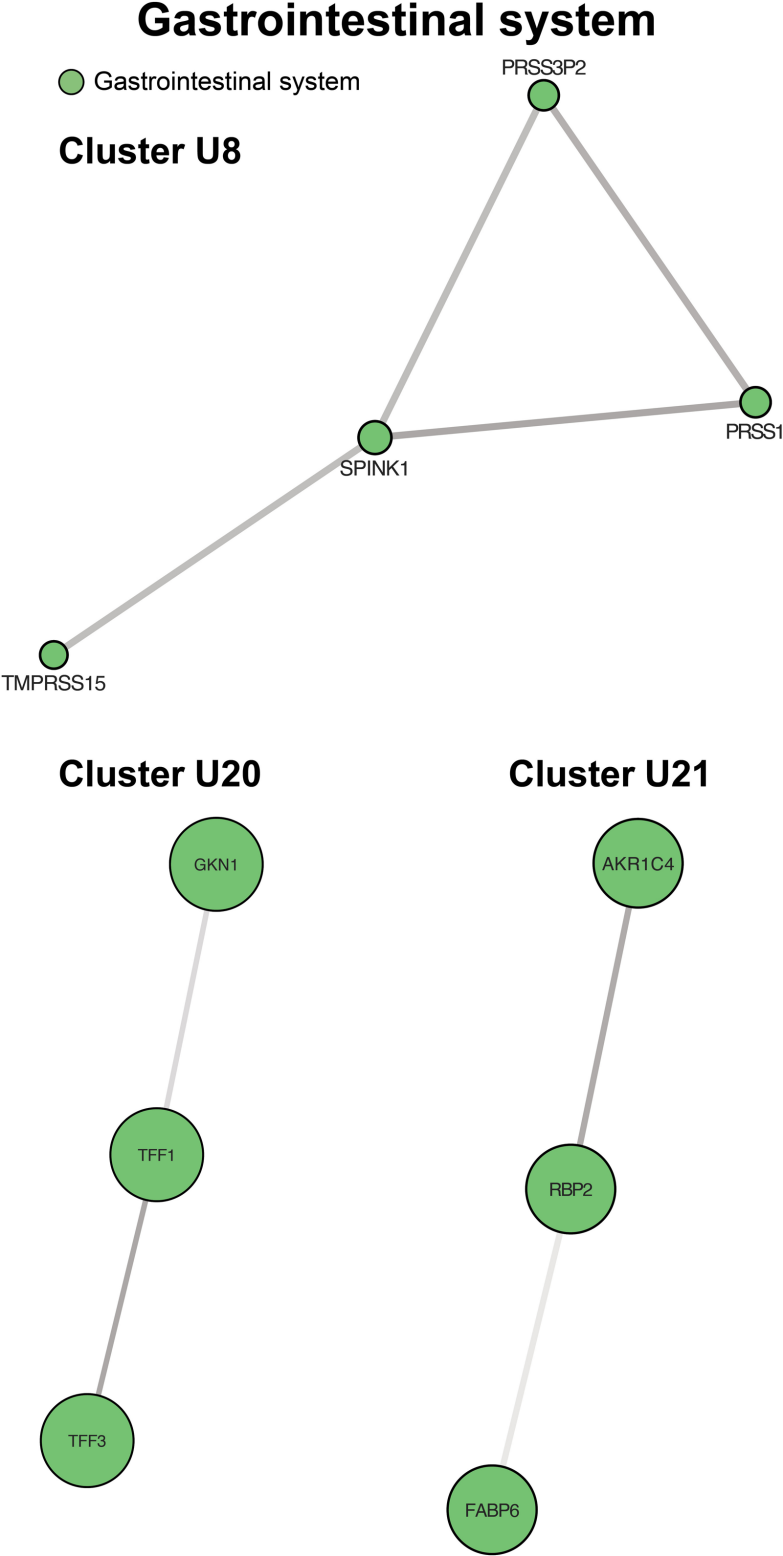
Clusters of gastrointestinal system processes. Cluster U8 contains genes involved in pancreatic inflammation. Cluster U20 centers on maintenance and protection of the gastrointestinal mucosa. Cluster U21 contains proteins controlling bile acid recycling. Cluster formatting is described in Figure 3.

Four up-regulated clusters involved the gastrointestinal system, mainly surrounding the intestinal mucosa, with functions in stabilizing the mucus gel overlying the gastrointestinal mucosa and promoting epithelial cell mobility to aid in healing (Figure 6, cluster U20). A second set of proteins bind bile acids and recycle them in ileal enterocytes (Figure 6, cluster U21). Finally, we identified two clusters involving pancreatic inflammation and function (Figure 6, cluster U8 and Figure 5, cluster D13).

### 3.8. Neural development

**Figure 7:**
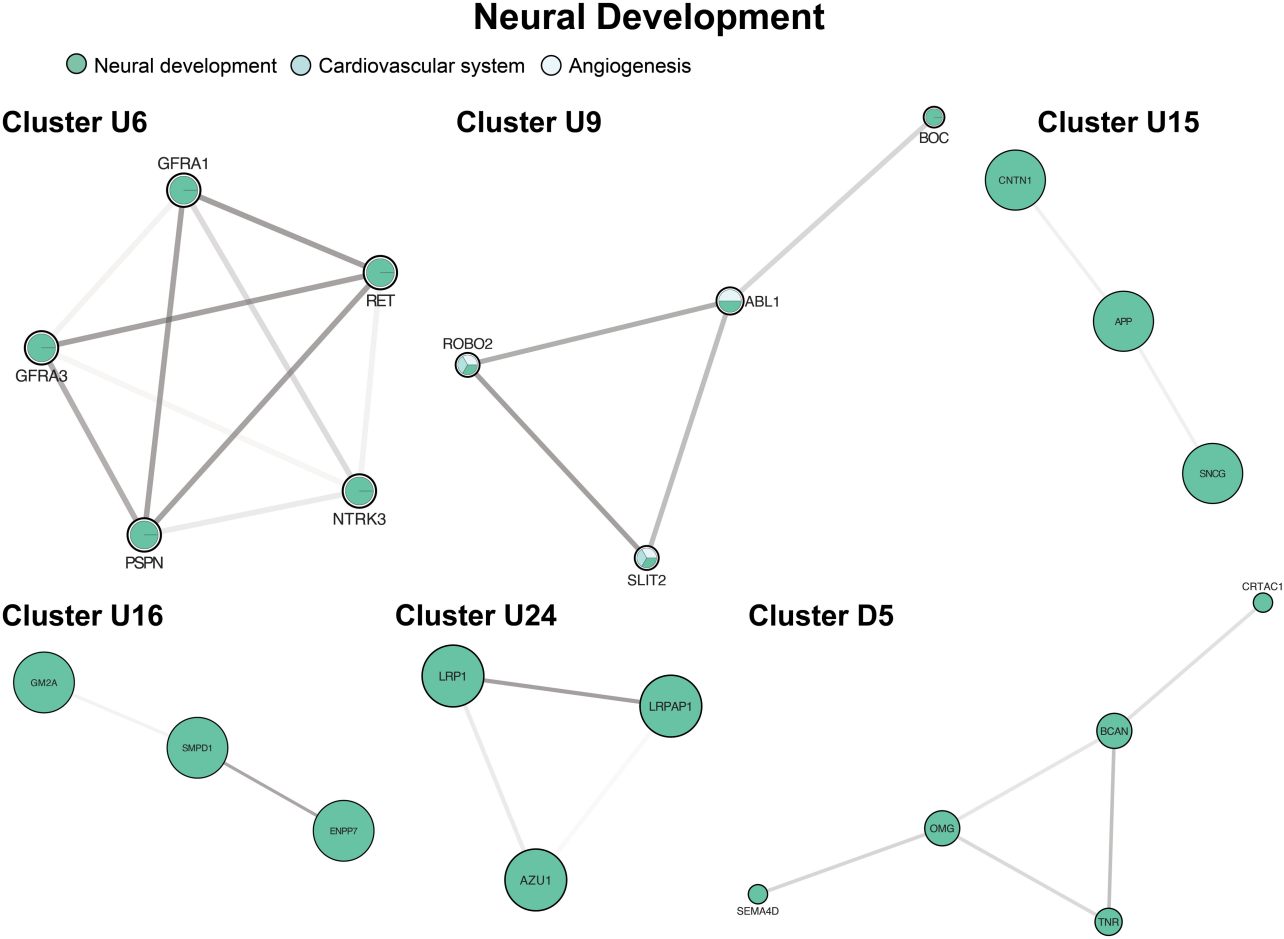
Clusters of neural development. Clusters U6, U9, U24, D5 involve drivers of axonogenesis and axon guidance. Cluster U15 contains individual proteins associated with cerebral plaque formation and autoimmune neuropathy Cluster U16 contains mediators of myelination. Cluster formatting is described in Figure 3.

Six unique clusters focused on processes within the nervous system (Figure 7, clusters U6, U9, U15, U16, U24, D5), and our largest cluster (U1a) was also highly enriched in neurologic processes. The most frequent processes in GO enrichment were axonogenesis and axon guidance with expression across both the central and peripheral nervous systems. We also observed multiple clusters involving axon myelination through sphingolipid metabolism (Figure 7, cluster U16) in the axonal myelin sheath and plasma membranes of the CNS. One cluster was not enriched by GO terms but contained individual proteins associated with cerebral plaque formation and autoimmune neuropathy (Figure 7, cluster U15).

### 3.9. Cardiovascular system

**Figure 8:**
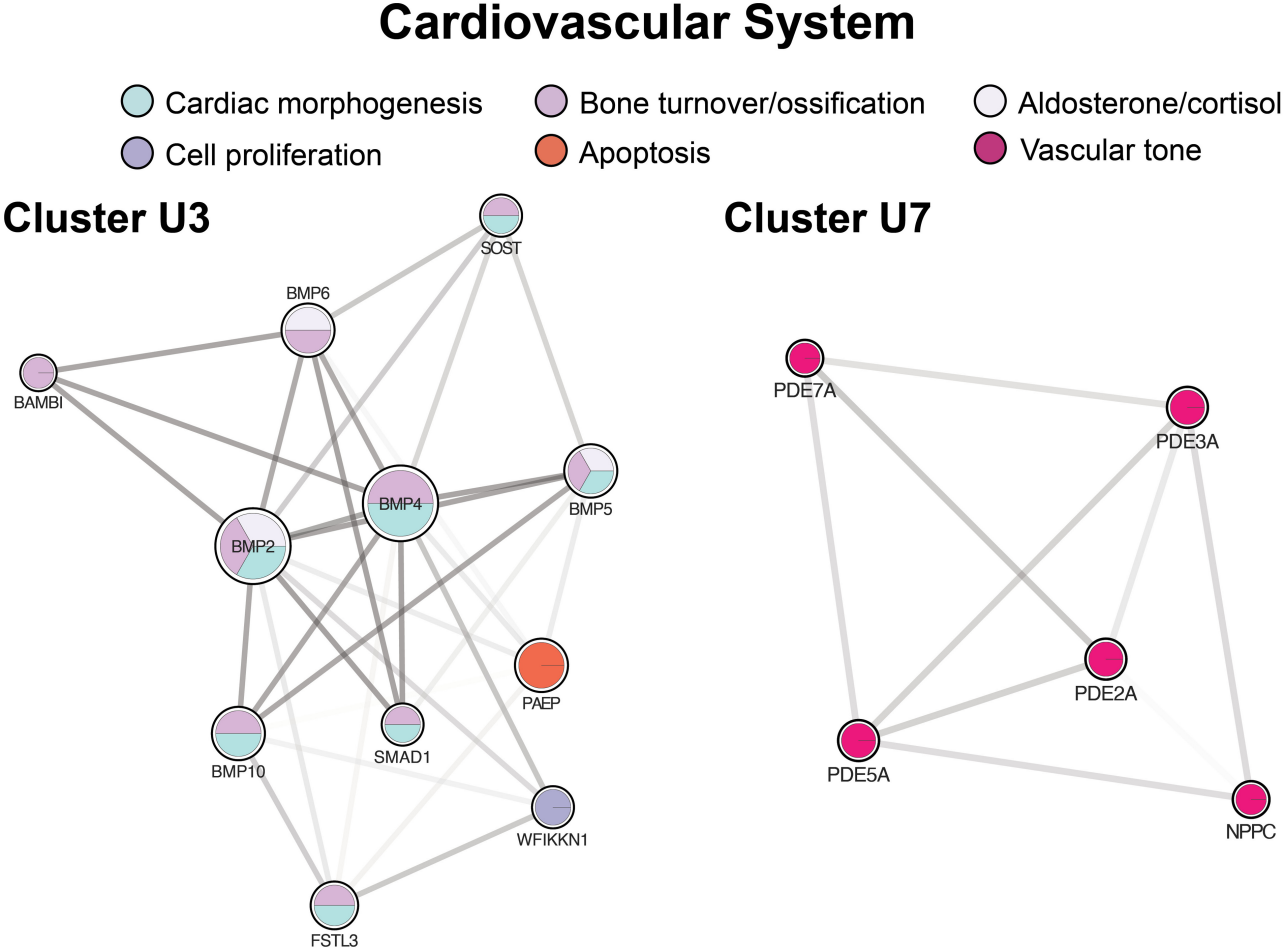
Clusters regulating the cardiovascular system. Cluster U3 centers of cardiac morphogenesis. Cluster U7 includes mediators of vascular tone. Cluster formatting is described in Figure 3.

The third largest up-regulated cluster (Figure 8, cluster U3) and a large fraction of clusters U1a and U9 concerned cardiac morphogenesis from vascular to valvular structures and cardiomyocyte proliferation and differentiation. Cluster U3 was also enriched in cardiac morphogenesis at the embryonic level through endocardial cushion development, forming the trabecular muscles, septum, and pericardium. Clusters U1a and U9 had additional proteins active during the embryonic period, contributing to forming the aortic, mitral, and pulmonic valves. Cluster U3 was also enriched in cortisol and aldosterone biosynthesis. Additional clusters mediated vascular tone through direct action on the vasculature and heart and through ion transport (Figure 8, cluster U7 and Figure 4, cluster D4).

## 4. Discussion

Single ventricle heart disease patients experience serious morbidity with clinical sequelae across organ systems. However, little is understood about the type and extent of molecular dysregulation SVHD patients experience. In this study, we analyzed serum to report a clear difference in the circulating proteomic fingerprint of infants with SVHD in the interstage period compared to similar-aged healthy controls. Furthermore, the proteome dysregulation they experience extends beyond cardiovascular regulation to include alterations in six primary areas: angiogenesis and cell proliferation/turnover, immune system regulation and inflammation, altered metabolism, gastrointestinal systems, neural development, and cardiac development.

### 4.1. Angiogenesis and cell proliferation/turnover

The largest and most highly interconnected cluster within our altered SVHD proteome centered on angiogenesis with functional enrichment of blood vessel formation across organs, including coronary vein and coronary artery morphogenesis, and glomerular capillary formation. One of the most potent drivers of angiogenesis, VEGFA, was at the center of this cluster. High levels of angiogenesis promoters in SVHD patients have been documented by others, including Dodge-Khatami et al., who reported elevated levels of VEGFA across the stages of SVHD palliation, peaking after Stage 2, followed by a slow decline until a nadir after completion of Stage 3 (24). In counterpoint, Bartoli et al. conducted a study similar to ours from samples drawn at the pre-Fontan catheterization and found levels of VEGFA to be downregulated compared to age matched-healthy controls despite up-regulation of numerous other pro-angiogenic factors (25). This discrepancy raises the question of whether the role of different angiogenic drivers may dominate at different stages of palliation. Further, we note that we cannot determine the origin of VEGFA from our circulating biomarker study, and consider it would be either systemic or pulmonary. In the systemic circulation, interstage physiology is marked by relative local tissue hypoxia secondary to arterial hypoxemia coupled with potentially compromised systemic cardiac output as the single ventricle pumps two effective cardiac outputs. The origin of VEGFA in this case could be systemic and hypoxia-driven, as local tissue hypoxia drives compensatory angiogenesis to overcome inadequate blood and oxygen supply (26). Interestingly, one of the most potent hypoxia-driven activators of VEGFA, HIF-1*α*, was not significantly different between our cases and controls, however, several other detectors of hypoxia, ENG and TGFB3, were. VEGFA may also have hypoxia-independent activation (27). The pulmonary endothelium experiences a marked shift in pressure and flow as the pulmonary vascular physiology transitions from a pulsatile flow (interstage) to a low-pressure, low-velocity continuous venous flow (Glenn). This discrepancy in pulmonary perfusion may explain both the increased VEGFA in our pre-Stage 2 cohort and the decreased VEGFA in the post-Stage 2 patients reported by the Bartoli group. Literature outside of CHD has shown changes in flow and pressure to directly alter protein synthesis at the pulmonary vascular endothelium, though not of angiogenic factors specifically (28). Notably, angiogenesis is not necessarily protective, as it also drives the growth of abnormal vasculature through the creation of systemic venous collaterals and multiple aortopulmonary collateral arteries (29).

We also demonstrated multiple fibroblast growth factors and metalloproteinases that function in tandem with VEGFA to achieve angiogenesis and other vascular changes. Proteins identified have roles in controlling the assembly and disassembly of the extracellular matrix (ECM) and collagen scaffolding, which support both the growth of new vessels and general cellular growth and repair. Notable proteins include FGF9 and FGF20, with roles in ECM changes in both the nervous and cardiovascular systems. Fibroblast growth factors have also been implicated as both a cause of pulmonary arterial hypertension (30, 31) as well as some evidence of, at times, protective effects (32). Our findings here are in alignment with our prior work and that of other groups in that SVHD patients have elevated circulating fibroblast growth factors, which may partly explain their proclivity to develop pulmonary hypertension (12, 33).

### 4.2. Immune system regulation and inflammation

Prior work by our group using a targeted cardiovascular protein panel demonstrated down-regulation of circulating pro-inflammatory cytokines within this cohort. Specific examples included interleukin (IL) 18, IL1 receptor ligand 2, tumor necrosis receptor superfamily member 13B, CXC motif chemokine 1, and leukocyte immunoglobulin-like receptor subfamily B1 (12). In our current study, we re-demonstrated lower levels of these specific proteins (with the exception of IL18 and leukocyte immunoglobulin-like receptor subfamily B1, which were not different between groups). There were a few other notable discrepancies between studies including discordant up vs. down regulation of ICAM3 and CD40LG (both up-regulated here). We suspect these differences to be secondary to differences and the relative quantification techniques across proteomics platforms. However, globally our expanded analysis includes additional members of the above immune pathways, demonstrating an overall trend toward up-regulation of pro-inflammatory cytokines. Included among the up-regulated proteins are proteins driving cytokine production, signaling, and response to cytokine activation. We also demonstrated an up-regulation of pro-apoptotic factors and a few instances of down-regulation of protective antioxidant factors. Inflammatory activation is well-described in post-surgical and post-bypass patients (34, 35), but our patients were, on average, 3–6 months out from initial palliative interventions. Therefore, the up-regulation of pro-inflammatory cytokines points to a broad chronic inflammatory state. The etiology of this is likely multifactorial with chronic poor oxygen delivery as a major mediator. Hypoxemia through chronic intermittent hypoxia (e.g., obstructive sleep apnea) has been shown to alter inflammatory mediators, including tumor necrosis factor (TNF)-α, interleukin (IL)-6 (36). Similarly, non-SVHD forms of chronic continuous hypoxemia (e.g., chronic obstructive pulmonary disease) have been shown to be altered at the microRNA level, though to a lesser degree than intermittent hypoxia (37). Our findings align with the results of studies of clinically available inflammatory markers that have identified elevated levels of inflammation in adults with CHD (38, 39). In adults, those with higher levels had worse functional status and exercise capacity and greater risk for death or non-elective cardiovascular hospitalization (40). The role and harm of inflammatory mediators in the pediatric CHD population will be an important area for ongoing investigation.

Immune regulators were expressed across both the up and down-regulated proteomes and across both the innate and adaptive immune systems. Changes in the adaptive immune system were more prominent, suggesting more chronic immune activation. We identified particular enrichment of mediators of T-cell activation, a phenomenon which has been well-described in other immune-activating chronic illnesses, including HIV and hepatitis C (41). Chronic immune activation is also associated with T-lymphocyte depletion (42, 43), consistent with described lymphopenia in many SVHD infants before both the stage 2 and stage 3 operations (44). A complicating factor in assessing the adaptive immune system alteration is the frequency of thymectomy in SVHD patients, as median sternotomy often requires the removal of the thymic tissue. Stages 1 and 2 typically occur during the first year of life, a critical period for thymic lymphocyte production (45, 46). Thymectomy has been associated with premature immunosenescence of peripheral T-cell populations both in the short- and long-term, effects that appear permanent in some studies (46, 47). There are also likely connections between the development of the cardiovascular and immune systems, which may cloud the assessment of immune function. Many of the most common genetic syndromes linked to (non-SVHD) CHD (e.g., Trisomy 21, DiGeorge syndrome, Noonan syndrome) have associated immune deficiencies or senescence, as well as increased risk of the malignancies of the hematopoietic system, affecting immunoregulatory cell populations (45, 48).

### 4.3. Altered metabolism

Altered cholesterol metabolism has previously been documented in hypoxia, secondary to oncologic processes with notable hepatic tissue hypoxia driving excessive hepatic steatosis (49) as well as lipogenesis, extracellular uptake of fatty acids, and lipogenesis (50). However, it should be noted that these are often more extreme forms of hypoxia, and the role of lower-grade chronic hypoxia on metabolism in CHD is not understood. Evidence of altered cholesterol metabolism has been identified directly in SVHD patients, though in the relatively normoxic post-Fontan period. In fact, these patients often experience metabolic dysregulation quite remotely, in many cases years out from the Fontan operation (51, 52). Specifically, post-Fontan patients have hypocholesterolemia, possibly due to hepatic insufficiency. Decreased cholesterol synthesis and resultant hypocholesterolemia is hypothesized as an early marker of liver disease, as it is associated with liver fibrosis even without correlative transaminases or gamma-glutamyl transpeptidase (GGT) elevation. To our knowledge, there has not been a study of cholesterol levels or synthesis earlier in the course of SVHD, nor the interstage period specifically. We found mixed results in proteins governing cholesterol synthesis. We demonstrated a down-regulation of numerous apolipoproteins, but circulating lipoproteins, including VLDL, LDL, and HDL, were well-represented in both up and down-regulated networks. We noted up-regulation of cholesterol and fat absorption markers with proteins driving bile acid binding and enterohepatic bile acid recycling (FABP6, AKR1C4), suggesting an overall increase in cholesterol and fat absorption capacity. This aligns with the findings of Whiteside et al., who showed an up-regulation of cholesterol absorption efficiency and low synthesis in Fontan patients (51). It has been posited that cholesterol absorption is up-regulated in an attempt to compensate for poor synthetic capacity in SVHD livers.

Additional metabolism pathways center on carbohydrate metabolism. First, several proteins central to carbohydrate digestion were down-regulated with low levels of amylases A1 and A2, though it is unclear how this may affect carbohydrate metabolism overall. The proteins trended slightly towards increased gluconeogenesis and decreased glycolysis, with a number of gluconeogenic proteins up-regulated (e.g., fructose-1,6-bisphosphatase and glucose-6-phosphate isomerase) and down-regulated proteins driving glycolysis (e.g., hexokinase 1 and 2, glucose-6-phosphate dehydrogenase). Prior metabolic evaluation of Fontan patients has shown no significant differences in glycolysis metabolites apart from lactate, which was elevated in Fontan patients when compared to healthy controls (53). Conclusions from our study are similarly limited as a number of proteins did not meet statistical significance, and a more complete mapping of gluconeogenic and glycolytic pathways is needed.

Additional protein derangements involving amino acid (e.g., tryptophan), nicotinate, and nicotinamide metabolism were noted and merit investigation, though outside the scope of this manuscript. Amino acid metabolism may be of particular interest, as we have previously demonstrated low circulating amino acids during the interstage period (54, 55).

### 4.4. Gastrointestinal systems

Regulators of the gastrointestinal system were well-represented, with particular emphasis on the intestines and pancreas. Perfusion insults to the intestines in the acute post-operative period result in high morbidity and, when severe, contribute to the development of necrotizing enterocolitis (56). Patients with HLHS, the most common diagnosis in our cohort, are at the highest risk of developing NEC among all congenital heart disease patients (10). In the sub-acute post-operative period, patients frequently experience feeding intolerance and growth failure (57) and in the long term, patients are at risk of developing protein-losing enteropathy (PLE). PLE is thought to occur secondary to alterations in mesenteric blood flow, systemic inflammation, neurohormonal activation, and protein glycosylation (58), many of which were demonstrated in our cohort. We identified up-regulation of a number of protective proteins. In particular, trefoil factors 1 and 3 (TFF1, TFF3) stabilize the mucus gel overlying the gastrointestinal mucosa, providing a physical barrier against noxious agents (59). TFF3 further supports the mucosal barrier by promoting the mobility of epithelial cells in the healing process (60). It is therefore conceivable that the up-regulation of these proteins represents an adaptive response to low levels of mesenteric ischemia and inflammation recognized in our other cluster sets.

There was also an up-regulation of various pancreatic enzymes, including endopeptidases, (e.g., trypsins 1 and 2), and carboxypeptidases (e.g., CPA2). In sum, this may imply an upregulation of protein digestion or markers of pancreatic hypoperfusion, inflammation, and/or injury. CPA2 has also been considered as a possible biomarker for pancreatic injury in rats, with preliminary studies showing a higher sensitivity of detection and of sustained increases in plasma observed over a longer time period when compared to clinically available biomarkers, lipase and amylase (61). It should be noted, however, that the difference in age along with likely differences in oral-motor skills between healthy controls and our SVHD cases may result in a difference in overall diet (e.g., primarily breast milk/formula diet vs. a diet including solid foods). As a difference in dietary intake may affect the proteome (62, 63), and particularly digestive enzymes, we are cautious to draw conclusions in this area without further studies

### 4.5. Neural development

Neurologic and neurodevelopmental impairment is well-documented in patients with SVHD, with as many as one-third of patients demonstrating moderate to severe impairment by IQ testing (64). Neurodevelopmental deficits occur across developmental domains, including fine and gross motor skills, language, and memory (8). Patients may also experience higher level deficits in executive function, social cognition, and impulse and emotional control (8, 65, 66). The causes are thought to be multifactorial, including shared genetic origins of fetal heart and brain development, hypoxemia beginning in the fetal period, chronically low cardiac output, ischemic injuries from cardiopulmonary bypass, injurious events peri-operatively (e.g., intracranial hemorrhage, seizure), and prolonged hospital stays resulting in increased noxious exposures and decreased developmental stimulation (67–69). Our network was highly enriched by up-regulated proteins clustering around processes within the nervous system. The highest degrees of enrichment were in axonogenesis, axon morphogenesis, and axon guidance enrichment across both the central and peripheral nervous systems, with notable processes including retinal ganglion axon guidance, olfactory bulb interneuron development, and motor neuron axon migration. Proteins within the contactin gene family (CNTN1, CNTN3, CNTN4, CNTN5) were nearly universally up-regulated. Notably, CNTN3 and CNTN5 were among the top 35 VIP proteins, along with NOTCH3 of the NOTCH signaling pathway, with which contactin acts as a ligand (70). The contactin family is an influential immunoglobulin superfamily with roles in axon outgrowth, guidance, and neuronal differentiation (71). The group has also been implicated in hippocampal synaptic plasticity and memory in adult mice (72).

Additionally, the network enrichment in CNS axon myelination through sphingolipid metabolism is of particular interest as myelination has been shown to correlate with intelligence with significant crossover in attributed genes (73). Further, delayed myelination is associated with developmental delay and behavioral impairments and in fact, long-term white matter alterations are a hallmark of brain imaging studies in SVHD patients (74). Although the cause is unknown, risk factors include perinatal inflammatory insults and fluctuating cerebral oxygenation on a background of chronic hypoxemia (75). The up-regulation of these processes in SVHD patients may therefore reflect a response to delayed myelination secondary to hypoxemic and inflammatory insults. However, our SVHD cohort skewed slightly younger than the healthy controls and thus perhaps experienced increased myelination owing to their younger age. Nevertheless, the myelination process should extend to at least two to three years of age, so both cohorts were well within the timeframe of active myelinating processes (76).

We were also interested in identifying biomarkers of neurologic injury. Prior work identified high levels of S100β, a glial-derived protein marker of cerebral ischemia, in HLHS patients (77, 78). Our protein panel did not test for S100β specifically but did target various other proteins in the S100 protein family and found similar elevation. The challenge with clinical translation of S100β has been the existence of extracerebral sources, confounding its utility as a marker of specific neurologic injury. It is unclear if similar proteins in the S100 family suffer from the same complication, but this would be an important area of investigation. Interestingly, commonly studied markers of neurologic injury were not similarly up-regulated. Specifically, GFAP was not significantly different between groups, and NEFL was down-regulated. These biomarkers peak with acute injurious events followed by a decline (on the order of months) and thus may not have been at chronically elevated levels even in patients with prior neurologic insults (79).

### 4.6. Cardiovascular system

We found mediation of the cardiovascular system at multiple levels, including cardiac morphogenesis, mediators of vascular tone and contractility through ion transport, and direct vasoactive proteins. Four key signaling pathways were well represented in the up-regulated protein network: NOTCH, TGFβ, insulin-like growth factor (IGF), and bone morphogenetic protein (BMP). NOTCH and BMP work synchronously in developing valvular structures such as the aortic and mitral valves (80–82). Together with TGFβ, they also drive cardiac morphogenesis at the embryonic level through endocardial cushion development, forming the trabecular muscles (BMP 5,10) and pericardium (BMP 2,5), among other structures (82, 83). In particular, NOTCH signaling is crucial for regulating a subset of endocardial cells in the primary heart tube (81). IGF, along with BMP, is responsible for cardiac growth through the proliferation of cardiomyocytes. Among this pathway, IGF2 has been identified as the most important mitogen for cardiomyocytes (84). Although our panel did not test for IGF2 itself, its receptor, as well as key binding proteins, were significantly higher in our SVHD cohort, suggesting activation of the pathway. Although the exact roles of proteins within these signaling pathways are still being elucidated, their general importance to the embryonic and fetal morphogenesis of the heart is understood. What is less clear is how these signaling pathways may be altered postnatally in patients with CHD and, further, how they may change at the various stages of intervention and beyond. Traditionally, cardiomyocytes were thought to be strictly postmitotic after birth, with further myocardial growth occurring by cell hypertrophy alone (85). However, growing work suggests possible cardiomyocyte regenerative ability at low levels. A number of genes and signaling pathways have been posited as potential drivers of regeneration. BMP, TGFβ, and NOTCH signaling have been identified as regenerative signaling pathways at sites of cardiomyocyte injury in non-mammalian animal models (86, 87). Mammalian studies have identified TNNI3K, a kinase interacting with TNNI3 (up-regulated in our cohort), as capable of cardiac stem cell dedifferentiation, conferring a regenerative capacity to the heart (88). The changes in these pathways and their possible roles in cardiomyocyte regeneration have not been well-studied in humans and, to our knowledge, not studied at all in CHD patients, specifically. SVHD and CHD patients may have abnormal circulating levels of these proteins at baseline, however, the broad up-regulation of several key signaling pathways in our SVHD patients raises the question of a possible role in cardiac growth and recovery in response to changing physiology.

BMP signaling is also central to GO enrichment of vascular tone mediators. Specifically, there was overall down-regulation (up-regulation of negative regulators) of aldosterone and cortisol, perhaps attributable to volume overload frequently experienced by interstage patients. Vasoactive proteins were enriched through negative regulators of transmembrane ion and cation (calcium) transport, affecting smooth muscle contraction. Interestingly, the same protein network demonstrated expression in striated muscle and sarcomeres and thus may have a role in cardiac contractility. Vascular permeability and resistance proteins were highly enriched through up-regulation of phosphodiesterase (PDE) signaling driving cGMP and cAMP catabolism. As cGMP and cAMP lead to vasodilation, up-regulation of PDEs causing increased catabolism of both has the end physiologic effect of vasoconstriction (89). This is deleterious in the pulmonary vasculature, where it is a primary cause of pulmonary hypertension. We demonstrated up-regulation of several phosphodiesterases, including PDE5, the therapeutic target of medical therapy for pulmonary hypertension, which works to inhibit PDE5, thereby increasing cGMP and vasodilation. Prior work has shown elevated PDE5 in SVHD patients with heart failure (90), but it is more commonly experienced following Stage 2 palliation where it is especially harmful as it hinders passive pulmonary blood flow. Our findings indicate that drivers of pulmonary hypertension may begin earlier in the course of palliation.

### 4.7. Additional enriched pathways

We identified several other areas of enrichment within our proteome that are outside the scope of this manuscript but may warrant future investigation. Most notable was a particularly high degree of bone ossification and bone turnover/resorption enrichment and, when considered alongside upregulated calcitriol metabolism, renal phosphate excretion, calcium transport, and calcium absorption, suggest an osteogenic focus.

### 4.8. Limitations

A limitation of this work is the difference in age and weight of our cases and controls. Although our inclusion criteria for age and weight were identical in both groups, patients in the control group tended to be undergoing elective and less time-sensitive surgical procedures. In standard clinical practice, this often leads to delaying procedures, which resulted in a skew of our control group to be slightly older and, therefore, heavier. To clarify the magnitude of this effect, we analyzed proteins against age and weight in our control group, who ranged from 5.12 to 11.66 months and 6.58–10.38 kg. Across these time and weight ranges, we found no effect of age or weight, which is reassuring against a large signal from either driving our observed differences in the proteomes. However, this does not preclude small age/weight effects, as it did not include the full age and weight range of all included subjects. The lack of studies on the effects of age on protein levels remains a gap in our field that merits dedicated investigation. We were unable to control for age or weight in our full analysis, as the case and control groups would split on that metric. Second, although our custom protein panel is a relatively large-scale assay, it still represents a subset of the total human proteome and does not include analysis of downstream proteome mediators (e.g., nucleic acids, metabolites, glycosylation, etc.). While the human proteome's exact size is unknown, estimates in the literature suggest there are approximately 20,000 total proteins (91–93). We therefore estimate that our targeted protein assay tests 7%–10% of *all* human proteins. It has been hypothesized that the detectable circulating proteome is smaller than the total proteome, but this value has not been quantified. Therefore, our study likely represents a higher fraction of proteins available in the blood. Additionally, the custom nature of our protein panel could introduce bias. To obviate this, we selected proteins of potential significance in cardiovascular, neurologic, and kidney function. In an exploratory effort, we also chose a variety of proteins of unknown significance to our patient population. An additional limitation is that our study does not identify a singular role of any one protein because many proteins tested are involved in multiple signaling pathways and function in more than one organ system. Further, individual proteins can be assigned to more than one GO term, introducing potential overlap with other functional groups. Finally, our data include a large number of proteins with *p* values between 0.05 and 1.1873e -13 between the various groups. Traditional Bonferroni correction is likely overly conservative and thus would underestimate the number of proteins affected during the interstage period (type 2 error). We therefore chose the less-conservative Benjamini-Hochberg (FDR) correction, and thus studies using absolute quantification methodologies in larger populations will be important to validate these results. Future work will also be needed to evaluate altered proteins’ changes over time and with subsequent interventions, including Stage 3 palliation and beyond. As our study shows multi-organ effects, evaluating these biological systems at the tissue level via an animal model will be necessary to understand mechanisms of dysregulation. Additionally, it will be important to collect clinical outcomes data for correlation and to begin to understand causation.

## 5. Conclusions

We report that the proteome of interstage SVHD subjects is readily distinguishable from healthy controls. This builds upon prior work, which demonstrated differences in the cardiovascular proteome in this cohort. Here, we present an expanded proteomic profile broadly dysregulated across multiple biological systems, including the nervous system, heart, vasculature, and gastrointestinal systems, with a high degree of activation of inflammatory cascades, underscoring the stress imposed by interstage SVHD physiology. Furthermore, this work demonstrates the degree of chronic protein dysregulation experienced by SVHD patients, even at a time of relative health within the course of palliation. This is an important step in understanding the sequelae of SVHD at the biochemical level, which is necessary to identify possible targets for prognostication and therapeutic intervention. In addition, the breadth of the biochemical changes highlights the importance of a systems-based approach in defining complex interactions not well captured by single biomarker strategies.

## Data Availability

The original contributions presented in the study are included in the article/supplementary materials, further inquiries can be directed to the corresponding author.
