# Supplementary material for: The proteomic fingerprint in infants with single ventricle heart disease in the interstage period: evidence of chronic inflammation and widespread activation of biological networks
Source: Front Pediatr. 2023 Dec 8;11:1308700. doi: 10.3389/fped.2023.1308700 (PMC10748388; doi:10.3389/fped.2023.1308700)
Supplement: Supplementary file 1 [file Datasheet1.zip › Datasheet1/Supplementary Files/Supplementary table 1.docx]

| **Custom Protein Panel** |
| --- |
| A2M, AADAT, AAMDC, ABHD14B, ABL1, ABRAXAS2, ACAA1, ACAN, ACE, ACE2, ACO1, ACOX1, ACP5, ACTN2, ACTN4, ACVRL1, ACY1, ACY3, ADA, ADA2, ADAM12, ADAM15, ADAM22, ADAM23, ADAM8, ADAM9, ADAMTS13, ADAMTSL2, ADCYAP1R1, ADGRB3, ADGRE5, ADGRF5, ADGRG2, ADH4, ADK, ADM, AFP, AGER, AGR2, AGRN, AGRP, AGT, AGXT, AHCY, AHSP, AIF1, AIF1L, AIFM1, AK1, AKR1A1, AKR1B1, AKR1B1, AKR1C4, AKT1S1, ALCAM, ALDH1A1, ALDH3A1, ALDOA, ALOX5, ALPI, ALPL, ALPP, AMBP, AMY2A, AMY2B, ANG, ANGPT1, ANGPT2, ANGPT4, ANGPTL1, ANGPTL1, ANGPTL3, ANGPTL4, ANGPTL7, ANGPTL8, ANK2, ANP32C, ANPEP, ANXA10, ANXA11, ANXA2, ANXA3, ANXA4, ANXA5, AOC1, AOC3, APEX1, APLP1, APOA1, APOA2, APOA4, APOA5, APOB, APOC1, APOC2, APOC3, APOD, APOE, APOF, APOH, APOL1, APOL2, APOL3, APOM, APP, APRT, AQP4, AREG, ARG1, ARG2, ARHGAP1, ARHGAP25, ARL2BP, ARNT, ARSA, ARSB, ART3, ASAH1, ASAH2, ASGR1, ASL, ASPN, ASRGL1, ATE1, ATOX1, ATP1B1, ATP1B2, ATP1B3, ATP5PO, ATP6V1F, ATXN10, AZU1, B3GAT3, B4GALT1, BACH1, BAG3, BAG6, BAMBI, BCAM, BCAN, BCL2L11, BDNF, BECN1, BGLAP, BGN, BHMT2, BID, BIN2, BIRC3, BLMH, BLVRB, BMP1, BMP10, BMP15, BMP2, BMP3, BMP4, BMP5, BMP6, BMP7, BMP8B, BMPR1A, BMPR1B, BMPR2, BOC, BOLA2, BPIFA2, BPIFB1, BRDT, BRK1, BSG, BST1, BST2, BTC, BTN3A2, C1GALT1C1, C1QA, C1QTNF1, C2, C2CD2L, C3, C4BPB, CA1, CA11, CA12, CA13, CA2, CA3, CA4, CA5A, CA6, CALB1, CALB2, CALCOCO2, CALY, CANT1, CAPG, CARHSP1, CASP10, CASP2, CASP3, CASP8, CASQ2, CBLIF, CBS, CBX2, CCDC80, CCL11, CCL14, CCL15, CCL16, CCL18, CCL19, CCL2, CCL20, CCL21, CCL22, CCL23, CCL24, CCL25, CCL27, CCL3, CCL5, CCL7, CCL8, CCN1, CCN2, CCN3, CCN4, CCN5, CCS, CCT5, CD109, CD14, CD14, CD160, CD163, CD177, CD2, CD200, CD209, CD22, CD248, CD27, CD274, CD2AP, CD300C, CD300LG, CD33, CD34, CD36, CD38, CD4, CD40LG, CD46, CD47, CD48, CD5, CD55, CD58, CD59, CD6, CD63, CD69, CD7, CD70, CD72, CD74, CD79B, CD80, CD83, CD8A, CD93, CD99L2, CDA, CDC37, CDCP1, CDH1, CDH15, CDH17, CDH2, CDH3, CDH5, CDH6, CDHR1, CDHR5, CDKN1A, CDKN2D, CDSN, CEACAM3, CEACAM8, CEBPB, CELA2A, CEP112, CEP43, CERT1, CES1, CETN2, CFC1, CGA, CGGBP1, CGREF1, CHEK2, CHGA, CHGB, CHI3L1, CHIT1, CHL1, CHMP1A, CHMP6, CHRDL2, CILP, CKAP4, CKB, CLC, CLDN1, CLEC10A, CLEC11A, CLEC1A, CLEC1B, CLEC4M, CLPP, CLPS, CLSTN1, CLTA, CLUL1, CNDP1, CNP, CNTN1, CNTN2, CNTN3, CNTN4, CNTN5, COCH, COL15A1, COL18A1, COL1A1, COL2A1, COL3A1, COL6A3, COL9A1, COMMD1, COMP, COMT, COPB2, COPE, COQ7, COX5B, CPA1, CPA2, CPB1, CPE, CPM, CPN2, CPPED1, CPQ, CPTP, CR2, CRADD, CREG1, CRH, CRHBP, CRIP2, CRKL, CRLF1, CRP, CRTAC1, CRTAM, CRYBB1, CRYZL1, CS, CSDE1, CSF1, CSF2, CSF2RA, CSF3, CSH1, CSPG4, CSRP3, CST3, CST5, CST6, CST7, CSTB, CTF1, CTHRC1, CTLA4, CTRB1, CTSB, CTSC, CTSD, CTSH, CTSO, CTSS, CTSV, CTSZ, CX3CL1, CXADR, CXCL1, CXCL10, CXCL11, CXCL12, CXCL13, CXCL14, CXCL16, CXCL3, CXCL5, CXCL6, CXCL8, CXCL9, CYTL1, DAB2, DAG1, DAP, DAPK1, DARS1, DARS2, DBI, DCN,, DCTN2, DCTN6, DCTPP1, DDAH1, DDR1, DDT, DDX58, DEFA1, DEFA5, DEFB4A, DFFA, DHODH, DIABLO, DKK1, DKK3, DKK4, DLK1, DLL1, DLL4, DLST, DNAJA2, DNAJB1, DNAJB8, DNPH1, DOK2, DPEP1, DPP4, DPP6, DPP7, DPT, DRAXIN, DSC2, DSG1, DSG2, DSG3, DSG4, DTYMK, DUSP3, EBAG9, EBI3, ECE1, ECHS1, ECI2, EDEM2, EDIL3, EDN1, EDN2, EDN3, EEF1A1, EEF1D, EFEMP1, EFNA1, EFNA4, EGF, EGFR, EHBP1, EHD3, EIF2S2, EIF4B, EIF4EBP1, EIF4G1, EIF5, EIF5A, ELANE, ENG, ENO1, ENO2, ENO3, ENOX2, ENPEP, ENPP2, ENPP6, NPP7, ENTPD5, ENTPD6, EPHA1, EPHA10, EPHA2, EPHB4, EPHB6, EPHX2, EPO, ERBB2, ERBB3, ERBB4, ERVV-1, ESAM, EZR, F11R, F3, F7, F9, FABP1, FABP2, FABP3, FABP4, FABP5, FABP6, FABP9, FADD, FAM160B1, FAM172A, FAM20A, FAM3B, FAM3C, FAP, FAS, FASLG, FBP1, FCAMR, FCAR, FCER2, FCGR2A, FCGR2B, FCGR3B, FCN2, FCRL1, FCRL5, FDX2, FEN1, FETUB, FGF1, FGF10, FGF12, FGF16, FGF17, FGF18, FGF19, FGF2, FGF20, FGF22, FGF23, FGF3, FGF4, FGF5, FGF6, FGF7, FGF8, FGF9, FGFBP1, FGFR1, FGFR2, FGR, FHIT, FIBP, FKBP1B, FKBP4, FKBP5, FKBP7, FLI1, FLRT2, FLT1, FLT3, FLT3LG, FLT4, FMR1, FOLR1, FOLR2, FOLR3, FOXM1, FOXO1, FOXO3, FRZB, FSHB, FST, FSTL1, FSTL3, FUCA1, FURIN, FUT3, FUT8, FXN, G6PD, GADD45GIP1, GAL, GALNT2, GALNT3, GAP43, GAPDH, GAS6, GATM, GBP2, GBP6, GCG, GCLM, GCNT1, GDF15, GDF2, GDNF, GET3, GFAP, GFER, GFRA1, GFRA2, GFRA3, GGA1, GGCT, GGH, GGT5, GH1, GH2, GHRL, GIP, GKN1, GLB1, GLO1, GLRX, GLT8D2, GM2A, GMFG, GMPR, GNLY, GNPDA1, GOLM2, GOT1, GOT2, GP1BA, GP1BB, GP5, GP6, GPC1, GPC5, GPI, GPNMB, GPR37, GRAP2, GRHPR, GRK5, GRN, GRP, GSS, GSTA1, GSTA3, GSTM4, GSTP1, GUK1, GUSB, GZMA, GZMB, GZMH, HAAO, HADH, HARS1, HAVCR2, HBQ1, HBZ, HCE000104, HCE000342, HCE000414, HCE000483, HCE001796, HCE003167, HCE003183, HCE003300, HCE004152, HCE004331, HCE004333, HCE004359, HCLS1, HDGF, HEBP1, HEPH, HEXIM1, HGF, HGS, HIF1A, HK1, HK2, HLA-E, HMBS, HMGB1, HMGB2, HMGCL,, , HMGCS1, HMOX1, HMOX2, HNMT, HNRNPK, HOXD4, HP, HPCAL1, HPGDS, HPRT1, HPSE, HRC, HS6ST1, HSD11B1, HSDL2, HSP90AA1, HSP90B1, HSPA1A, HSPB1, HSPB6, HSPG2, HTRA2, HYAL1, HYOU1, ICA1, ICAM1, ICAM2, ICAM3, ICAM4, ICAM5, ICOSLG, IDH1, IDI2, IDO1, IDS, IDUA, IFNG, IFNGR1, IFNGR2, IFNL1, IFNW1, IGDCC4, IGF1, IGF1R, IGF2R, IGFBP1, IGFBP2, IGFBP3, IGFBP4, IGFBP6, IGFBP7, IGFBPL1, IGSF3, IGSF8, IL10, IL10RB, IL11, IL12A, IL12B, IL12B, IL12RB1, IL13, IL13RA1, IL15, IL15RA, IL16, IL17A, IL17RA, IL18, IL18BP, IL18R1, IL18RAP, IL19, IL1A, IL1B, IL1R1, IL1R2, IL1RAP, IL1RL1, IL1RN, IL2, IL20, IL21, IL22, IL24, IL26, IL27, IL2RA, IL2RB, IL3, IL32, IL34, IL3RA, IL4, IL4R, IL5, IL5RA, IL6, IL6, IL6R, IL6ST, IL7, IL7R, IL9, ILKAP, IMPA1, ING1, INHBC, INPP5D, IRAG2, ISLR2, ITGA5, ITGA6, ITGAL, ITGAM, ITGAV, ITGB1, ITGB1BP2, ITGB2, ITGB5, ITGB6, ITGB7, ITIH3, ITM2A, ITPA, IVD, JAM2, JCHAIN, JUN, KCNE5, KCNIP4, KDR, KEL, KHK, KIF1C, KIF22, KIR2DL3, KIR3DL1, KIRREL2, KIT, KITLG, KL, KLB, KLK10, KLK3, KLK8, KLRB1, KMO, KRT14, KRT17, KRT18, KRT19, KRT5, KYAT1, KYAT3, KYNU, L1CAM, L3HYPDH, LACTB2, LAG3, LAIR2, LAMA4, LAMP1, LAP3, LAT, LAYN, LBP, LCN1, LCN2, LCP1, LDLR, LECT2, LEFTY2, LEP, LEPR, LGALS1, LGALS2, LGALS3, LGALS3BP, LGALS4, LGALS7, LGALS8, LGALS9, LGALSL, LHB, LIF, LIFR, LILRA2, LILRA3, LILRA4, LILRA5, LILRA6, LILRB1, LILRB2, LILRB5, LMOD1, LONP1, LPL, LPO, LRCH4, LRCH4, LRP1, LRP11, LRPAP1, LSM1, LSP1, LTA, LTA, LTA4H, LTBP4, LTBR, LXN, LY6D, LY75, LYN, LYPD1, LYPD3, LYVE1, MAD1L1, MAMDC2, MAN1A2, MAN2B2, MANF, MANSC4, MAP1LC3B2, MAP2K6, MAP4K5, MAPK9, MAPKAPK2, MAPT, MARCO, MASP1, MAT1A, MAT2A, MATN2, MATN3, MAX, MB, MCAM, MDGA1, MDH1, MDH2, MDK, MEP1A, MERTK, MESD, MET, METAP1, METAP2, MFAP3, MFAP5, MFGE8, MGMT, MIA, MIF, MILR1, MITD1, MLN, MME, MMP1, MMP10, MMP12, MMP13, MMP14, MMP16, MMP17, MMP19, MMP2, MMP20, MMP3, MMP7, MMP8, MMP9, MNDA, MOG, MPI, MPIG6B, MPO, MSLN, MSMB, MSR1, MSTN, MTAP, MVK, MYBPC1, MYBPC2, MYDGF, MYL12B, MYL3, MYL4, MYOC, MYOM2, NAA10, NAAA, NADK, NADK2, NAGK, NAMPT, NARS1, NBL1, NCAM1, NCAM2, NCAN, NCF2, NCK2, NCR1, NDRG1, NDUFA2, NDUFAF5, NDUFB11, NECAP2, NECTIN2, NEFL, NFASC, NFATC1, NFKB1, NFKBIA, NFKBIE, NFYA, NGF, NGFR, NID1, NID2, NIT2, NMNAT1, NMRK1, NMRK2, NMT1, NNMT, None, NOS3, NOSIP, NOTCH1, NOTCH2, NOTCH3, NPDC1, NPL, NPM1, NPPA, NPPB, NPPB, NPPB, NPPC, NPTX1, NPTX2, NPTXR, NPY, NQO1, NR3C2, NRCAM, NRP1, NRP2, NSFL1C, NT5C, NT5E, NTF3, NTF4, NTRK2, NTRK3, NUCB2, NUDT1, NUDT10, NUDT15, NUDT2, NUDT5, NXPH1, OAT, OAZ1, OBP2B, ODC1, OGN, OLR1, OMG, OSM, OSMR, OXCT1, OXT, P4HB, PADI4, PAEP, PAFAH1B2, PAFAH1B3, PAFAH2, PAGR1, PAK4, PAM, PAPPA, PARK7, PARP1, PC, PCDH1, PCDH17, PCK1, PCK2, PCOLCE, PCSK1, PCSK9, PDAP1, PDCD1, PDCD5, PDCD6, PDE2A, PDE3A, PDE4D, PDE5A, PDE7A, PDE9A, PDGFA, PDGFB, PDGFC, PDGFD, PDGFRA, PDGFRB, PDGFRL, PDIA4, PEAR1, PEBP1, PECAM1, PECR, PF4, PF4V1, PFDN2, PFKM, PGAM1, PGD, PGF, PGK1, PGLYRP4, PGM1, PHOSPHO1, PI3, PIGR, PIK3IP1, PILRA, PKD2, PKLR, PKM, PLA2G10, PLA2G1B, PLA2G2A, PLA2G4A, PLA2G7, PLAT, PLAU, PLAUR, PLG, PLIN3, PLPBP, PLTP, PLXNB2, PLXNB3, PMM2, PMVK, PNLIPRP2, POLR2F, POMC, PON1, PON2, POR, POSTN, PPARG, PPCDC, PPCS, PPIB, PPIF, PPM1A, PPM1F, PPP1R2, PPP3R1, PPY, PQBP1, PRCP, PRDX1, PRDX3, PRDX5, PRDX6, PRG2, PRG3, PRKAR1A, PRKCQ, PRKG1, PRKRA, PRL, PROC, PROCR, PROK1, PROS1, PRSS1, PRSS2, PRSS27, PRSS8, PRTFDC1, PRTN3, PSAP, PSG1, PSG7, PSIP1, PSMA1, PSMD9, PSME1, PSME2, PSPN, PTEN, PTGDS, PTGR1, PTGR2, PTGS2, PTH1R, PTK7, PTN, PTPN1, PTPN6, PTPRS, PTS, PTX3, PVR, PYDC1, PYY, QDPR, QPCT, QPRT, RAB11FIP3, RAB27B, RAB33A, RAB39B, RAB6A, RAB6B, RAD23B, RANBP1, RANGAP1, RARRES1, RARRES2, RASA1, RASSF2, RBKS, RBM19, RBP2, RBP5, RBPMS2, REG1A, REG1B, REG3A, RELT, REN, RET, RETN, REXO2, RGMA, RGMB, RGS8, RHOC, RILPL2, RLN3, RNASE10, RNASE3, RNF149, ROBO2, ROBO4, ROR1, RP2, RPS10, RRM2, RSPO1, RSRP1, RWDD1, S100A11, S100A12, S100A16, S100A4, S100A5, S100P, SARG, SAT1, SBSN, SCARA5, SCARB1, SCARB2, SCARF1, SCARF2, SCGN, SCN4B, SCP2, SCPEP1, SCT, SDHAF1, SDHB, SELE, SELL, SELP, SELPLG, SEMA4D, SEMA7A, SERPINA1, SERPINA11, SERPINA12, SERPINA5, SERPINB1, SERPINB5, SERPINB8, SERPINB9, SERPINC1, SERPINE1, SETMAR, SF3B4, SFN, SFRP1, SFTPB, SFTPD, SGSH, SH2D1A, SHD, SHMT1, SIGLEC15, SIGLEC5, SIGLEC6, SIGLEC7, SIL1, SIRPA, SIRPB1, SIRT1, SIRT2, SIRT3, SIRT5, SLAMF1, SLIT2, SLITRK6, SMAD1, SMPD1, SNAP23, SNAP29, SNCG, SNU13, SNX5, SOD1, SOD2, SOD3, SORD, SORT1, SOST, SPARC, SPARCL1, SPINK1, SPINK4, SPINK5, SPINK8, SPINT1, SPINT2, SPOCK1, SPON2, SPP1, SRC, SRP14, SRPK2, SSB, ST3GAL1, ST6GAL1, STAB2, STAMBP, STAT3, STAT5B, STC1, STC2, STIP1, STK24, STK4, STX4, STX6, SULT1A1, SULT2A1, SUMF2, SUSD1, SYN3, SYTL4, TACSTD2, TAGLN2, TALDO1, TARBP2, TBCB, TBCC, TCL1A, TCL1B, TCN2, TDGF1, TDO2, TEF, TEK, TFF1, TFF2, TFF3, TFPI, TFPI2, TFRC, TGFB1, TGFB3, TGFBI, TGFBR1, TGFBR2, TGFBR3, TGM2, THBS2, THBS4, THOP1, THPO, THY1, TIA1, TIE1, TIGAR, TIMD4, TIMP1, TIMP2, TIMP3, TIMP4, TINAGL1, TJP1, TLR2, TLR3, TLR4, TLR5, TMED10, TMED4, TMEM132A, TMPRSS15, TMPRSS5, TNC, TNF, TNFAIP8, TNFRSF10A, TNFRSF10B, TNFRSF11B, TNFRSF13B, TNFRSF1A, TNFRSF1B, TNFRSF21, TNFRSF4, TNFRSF6B, TNFRSF8, TNFSF10, TNFSF11, TNFSF12, TNFSF13B, TNFSF14, TNFSF8, TNNI2, TNNI3, TNNT2, TNNT3, TNR, TNXB, TOMM20, TOR1AIP1, TP53, TPH1, TPK1, TPM3, TPMT, TPP1, TPPP3, TPSAB1, TPT1, TREML2, TRIAP1, TRIM21, TSLP, TST, TTN, TWF2, TXLNA, TXNDC5, TXNRD1, TYMP, TYRO3, UBQLN3, UGDH, ULBP2, UMOD, UPB1, USO1, USP8, VASN, VCAM1, VCAN, VCL, VEGFA, VEGFA, VEGFB, VEGFC, VEGFD, VIL1, VIM, VIP, VIT, VNN2, VSIG10L, VSIG4, VSIR, VSTM1, VSTM2L, VTA1, VWC2, VWF, WARS1, WAS, WFDC2, WFIKKN1, WWP2, XCL1, XDH, XG, XRCC4, YES1, ZADH2, ZBTB16 |

**Supplementary Table 1.** Complete list of proteins tested in the SomaLogic assay, listed in alphabetical order.
