# Supplementary material for: The proteomic fingerprint in infants with single ventricle heart disease in the interstage period: evidence of chronic inflammation and widespread activation of biological networks
Source: Front Pediatr. 2023 Dec 8;11:1308700. doi: 10.3389/fped.2023.1308700 (PMC10748388; doi:10.3389/fped.2023.1308700)
Supplement: Supplementary file 1 [file Datasheet1.zip › Datasheet1/Supplementary Files/Supplementary table 2.docx]

| **GO term** | **GO number** | **FDR** | **Summary group** | **Cluster** |
| --- | --- | --- | --- | --- |
| Angiogenesis, blood vessel branching | GO:0001525 | 1.14E-27 | Angiogenesis | U1a |
| Connective tissue response to inflammation, wound healing | GO:0042060 | 1.69E-08 | Cell proliferation and turnover | U1a |
| Response to and detection of hypoxia | GO:0001666 | 4.73E-07 | Angiogenesis | U1a |
| Positive regulation of cell proliferation by VEGF, FGF | GO:0038091 | 8.22E-05 | Angiogenesis, Cell proliferation | U1a |
| Metanephric and glomerular capillary formation | GO:0072104 | 0.0003 | Angiogenesis, Kidney development | U1a |
| Cardiac morphogenesis, coronary artery, pericardium dev. | GO:1901652 | 0.00046 | Cardiovascular | U1a |
| Neural crest cell migration and projection ANS development | GO:0070570 | 0.00053 | Neural development | U1a |
| Glomerular mesangium development | GO:0072109 | 0.019 | Kidney development | U1a |
| Extracellular matrix organization and disassembly | GO:0030198 | 0.00012 | Cell proliferation and turnover | U1b |
| Collagen formation | GO:0030574 | 0.00078 | Cell proliferation and turnover | U1b |
| Fibroblast growth factors | GO:0008543 | 1.87E-08 | Cell proliferation and turnover | U1c |
| Protein kinase | GO:0051897 | 1.73E-07 | Cell proliferation and turnover | U1c |
| MAPK cascade | GO:0000165 | 5.39E-06 | Cell proliferation and turnover | U1c |
| Cardiac muscle cell proliferation | GO:0060043 | 0.0213 | Cardiovascular | U1c |
| Extracellular matrix | GO:0031012 | 0.0336 | Cell proliferation and turnover | U1d |
| Cellular response to cytokine stimulus | GO:0034097 | 8.28E-32 | Inflammation | U2 |
| Inflammatory response | GO:0006954 | 9.23E-22 | Inflammation | U2 |
| Positive regulation of leukocyte cell-cell adhesion | GO:1903039 | 4.76E-19 | Adaptive immune system | U2 |
| Positive regulation of T-cell activation | GO:0050870 | 8.78E-17 | Adaptive immune system | U2 |
| Leukocyte chemotaxis | GO:0030595 | 1.13E-14 | Adaptive immune system | U2 |
| Cellular response to interleukins | GO:0070555 | 2.93E-14 | Inflammation | U2 |
| Innate immune response | GO:0045087 | 4.68E-11 | Innate immune system | U2 |
| Response to interferon-gamma | GO:0034341 | 8.97E-11 | Innate immune system | U2 |
| Serine/threonine kinase signaling pathway | GO:0090092 | 6.03E-12 | Cell proliferation/turnover | U3 |
| SMAD protein signal transduction | GO:0060395 | 3.10E-09 | Cell proliferation/turnover | U3 |
| BMP signaling and regulation | GO:0030509 | 1.73E-08 | Cardiovascular, Bone turnover | U3 |
| Ossification and cartilage development | GO:0001503 | 4.68E-08 | Bone turnover/ossification | U3 |
| Negative regulation of aldosterone biosynthetic process | GO:0032347 | 9.67E-06 | Cardiovascular | U3 |
| Negative regulation of cortisol biosynthetic process | GO:2000065 | 0.00058 | Cardiovascular | U3 |
| Bone morphogenic (BMP) signaling and regulation | GO:0030510 | 0.0015 | Cardiovascular | U3 |
| Cholesterol metabolism | GO:0008203 | 1.55E-05 | Lipid metabolism | U4 |
| Chylomicron remodeling, assembly | GO:0034382 | 0.0031 | Lipid metabolism | U4 |
| Plasma lipoprotein particle assembly, remodeling, clearance | GO:0034377 | 0.00035 | Lipid metabolism | U4 |
| Phospholipid metabolism, efflux | GO:0006644 | 0.0044 | Lipid metabolism | U4 |
| VLDL lipoprotein particle assembly | GO:0034379 | 0.0047 | Lipid metabolism | U4 |
| LDL lipoprotein particle assembly | GO:0034374 | 0.0059 | Lipid metabolism | U4 |
| HDL lipoprotein particle assembly | GO:0034375 | 0.0075 | Lipid metabolism | U4 |
| Monosaccharide biosynthesis and metabolism | GO:0005996 | 3.03E-05 | Carbohydrate metabolism | U5 |
| Pentose-phosphate shunt and metabolism | GO:0006098 | 0.0246 | Carbohydrate metabolism | U5 |
| Hexose metabolism | GO:0019318 | 0.0273 | Carbohydrate metabolism | U5 |
| Glial cell neurotrophic factor receptor signaling pathway | GO:0035860 | 2.26E-05 | Neural development | U6 |
| Axon guidance | GO:0007411 | 0.0013 | Neural development | U6 |
| MAPK cascade | GO:0000165 | 0.0014 | Neural development | U6 |
| Autonomic nervous system development | GO:0048483 | 0.0203 | Neural development | U6 |
| Peripheral nervous system development | GO:0007422 | 0.0487 | Neural development | U6 |
| cGMP metabolism and mediation | GO:0046068 | 2.34E-05 | Vascular tone | U7 |
| Positive regulation of vascular permeability | GO:0043117 | 0.0032 | Vascular tone | U7 |
| Negative regulation of vascular permeability | GO:0043116 | 0.0036 | Vascular tone | U7 |
| Negative regulation of cAMP-mediated signaling | GO:0043951 | 0.0056 | Vascular tone | U7 |
| Pancreatic endocrine function, secretory enzymes | GO:0031018 | 0.0026 | Gastrointestinal system | U8 |
| Serine-type endopeptidase activity | GO:0004252 | 0.0065 | Gastrointestinal system | U8 |
| Olfactory bulb interneuron development | GO:0021891 | 0.002 | Neural development | U9 |
| Axonogenesis | GO:0007409 | 0.002 | Neural development | U9 |
| Aortic and pulmonic valve, ventricular septum morphogenesis | GO:0003184 | 0.0034 | Cardiovascular | U9 |
| Retinal ganglion axon guidance | GO:0003184 | 0.0036 | Neural development | U9 |
| Axon guidance | GO:0007411 | 0.0056 | Neural development | U9 |
| Circulatory system development | GO:0072359 | 0.0481 | Cardiovascular | U9 |
| O-glycan processing | GO:0016266 | 0.0003 | Protein sugar modifications | U10 |
| Protein glycosylation | GO:0006486 | 0.0003 | Protein sugar modification | U10 |
| No significant enrichment |  |  | Ungrouped | U11 |
| No significant enrichment |  |  | Ungrouped | U12 |
| ATM signaling pathway: DNA damage repair, cell cycle arrest | GO:0023052 | 0.0087 | Apoptosis | U13 |
| Apoptosis | GO:0042981 | 0.0135 | Apoptosis | U13 |
| Nodal signaling pathway: cell differentiation | GO:0038092 | 0.0131 | Cell proliferation and turnover | U14 |
| Serine/threonine kinase receptor pathway | GO:0007178 | 0.0131 | Cell proliferation and turnover | U14 |
| No significant enrichment |  |  | Ungrouped | U15 |
| Sphingolipid metabolism | GO:0030149 | 5.10E-05 | Neural development | U16 |
| Glycosphingolipid metabolism | GO:0006687 | 0.00015 | Neural development | U16 |
| Sphingomyelin catabolism | GO:0006685 | 0.00094 | Neural development | U16 |
| No significant enrichment |  |  | Ungrouped | U17 |
| No significant enrichment |  |  | Ungrouped | U18 |
| Fatty acid beta oxidation | GO:0033540 | 0.0133 | Lipid metabolism | U19 |
| Lipid catabolism | GO:0044242 | 0.0133 | Lipid metabolism | U19 |
| Alpha-linolenic acid metabolism | GO:0036109 | 0.0133 | Lipid metabolism | U19 |
| Regulation of lipid metabolism | GO:0019216 | 0.0265 | Lipid metabolism | U19 |
| Digestion | GO:0007586 | 0.0026 | Gastrointestinal system | U20 |
| Maintenance of gastrointestinal epithelium | GO:0030277 | 0.0232 | Gastrointestinal system | U20 |
| Retinoid metabolism and transport | GO:0194068 | 0.0336 | Gastrointestinal system | U21 |
| Bile acid and bile salt metabolism | GO:0006699 | 0.0336 | Gastrointestinal system | U21 |
| Positive regulation of bone resorption | GO:0042572 | 0.0423 | Bone turnover/ossification | U22 |
| Osteoclast differentiation | GO:0030316 | 0.0423 | Bone turnover/ossification | U22 |
| Transferrin endocytosis and recycling |  | 0.0423 | Ungrouped | U22 |
| Renal phosphate excretion | GO:0000107 | 0.0015 | Kidney function | U23 |
| Positive regulation of amyloid-beta clearance | GO:1900223 | 0.0072 | Neural development | U24 |
| Protein kinase C-activating G-protein coupled receptor signal | GO:0007205 | 0.0454 | Neural development | U24 |
| Glial cell activation | GO:0061900 | 0.0454 | Neural development | U24 |
| Desmosome integrity | GO:0030057 | 0.0093 | Cell proliferation and turnover | U25 |
| Cornified envelope | GO:0001533 | 0.0144 | Cell proliferation and turnover | U25 |

**Supplementary Table 2.** Complete list of enriched up-regulated GO terms and their associated GO number, summary group, and cluster, organized from smallest to largest FDR. Color coding correlates with summary group coloring as seen in the cluster figures within the primary manuscript.
