# Supplementary material for: The proteomic fingerprint in infants with single ventricle heart disease in the interstage period: evidence of chronic inflammation and widespread activation of biological networks
Source: Front Pediatr. 2023 Dec 8;11:1308700. doi: 10.3389/fped.2023.1308700 (PMC10748388; doi:10.3389/fped.2023.1308700)
Supplement: Supplementary file 1 [file Datasheet1.zip › Datasheet1/Supplementary Files/Supplementary table 3.docx]

| **GO term** | **GO number** | **FDR** | **Summary group** | **Cluster** |
| --- | --- | --- | --- | --- |
| Regulation of immune system processes | GO:0002376 | 1.11E-17 | Immune system | D1 |
| Cytokine-mediated signaling pathway, cytokine production | GO:0019221 | 5.44E-15 | Inflammation | D1 |
| Leukocyte activation, differentiation | GO:0045321 | 8.13E-09 | Adaptive immune system | D1 |
| Adaptive immune response | GO:0002250 | 2.27E-08 | Adaptive immune system | D1 |
| Innate immune response | GO:0045087 | 1.09E-05 | Innate immune system | D1 |
| Regulation of cell death | GO:0010941 | 1.29E-05 | Apoptosis | D1 |
| Nicotinate and nicotinamide metabolism | GO:0015664 | 0.00012 | Metabolism | D1 |
| Positive regulation of calcidiol 1-monooxygenase activity | GO:0060559 | 0.004 | Metabolism | D1 |
| Positive regulation of superoxide dismutase activity | GO:1901671 | 0.0056 | Response to cell injury | D1 |
| Ion transport | GO:1904062 | 0.00015 | Cardiovascular, vascular tone | D2 |
| Actin filament-based cell movement | GO:0030048 | 0.0013 | Cardiovascular | D2 |
| Smooth muscle contraction | GO:0006936 | 0.0151 | Cardiovascular, vascular tone | D2 |
| Lipid transport | GO:0006869 | 1.52E-16 | Lipid metabolism | D3 |
| Cholesterol transport | GO:0030301 | 2.80E-12 | Lipid metabolism | D3 |
| HDL lipoprotein particle remodeling | GO:0034375 | 5.93E-10 | Lipid metabolism | D3 |
| Triglyceride homeostasis, catabolism, metabolism | GO:0070328 | 1.33E-09 | Lipid metabolism | D3 |
| VLDL particle remodeling | GO:0034372 | 1.90E-08 | Lipid metabolism | D3 |
| Positive regulation of fatty acid biosynthesis | GO:0045723 | 2.07E-07 | Lipid metabolism | D3 |
| Chylomicron assembly, remodeling | GO:0034371 | 6.31E-06 | Lipid metabolism | D3 |
| Steroid biosynthesis | GO:0006694 | 0.00011 | Lipid metabolism | D3 |
| Regulation of systemic arterial blood pressure | GO:0070555 | 0.0282 | Cardiovascular | D4 |
| Regulation of lymphocyte activation | GO:0045087 | 0.0282 | Adaptive immune system | D4 |
| Regulation of immune system processes | GO:0002682 | 0.0404 | Immune system | D4 |
| Negative regulation of axonogenesis | GO:0050771 | 0.0064 | Neural development | D5 |
| Neuron projection development | GO:0031175 | 0.0231 | Neural development | D5 |
| Telencephalon development | GO:0021537 | 0.0324 | Neural development | D5 |
| Nervous system development | GO:0007399 | 0.0324 | Neural development | D5 |
| Cell adhesion | GO:0007155 | 0.0324 | Cell proliferation/turnover | D5 |
| Negative regulation of axon extension | GO:0030517 | 0.0367 | Neural development | D5 |
| No significant enrichment |  |  | Ungrouped | D6 |
| Canonical glycolysis | GO:0061621 | 0.00017 | Carbohydrate metabolism | D7 |
| Carbohydrate metabolism | GO:0005975 | 0.00019 | Carbohydrate metabolism | D7 |
| Fructose 6-phosphate metabolism | GO:0006002 | 0.0013 | Carbohydrate metabolism | D7 |
| No significant enrichment |  |  | Ungrouped | D8 |
| No significant enrichment |  |  | Ungrouped | D9 |
| Cysteine and methionine metabolism | GO:0006534 | 5.95E-06 | Amino acid metabolism | D10 |
| Sulfur amino acid biosynthesis | GO:0000097 | 0.0172 | Amino acid metabolism | D10 |
| No significant enrichment |  |  | Ungrouped | D11 |
| Kynurenine-oxoglutarate transaminase activity | GO:0016212 | 0.00076 | Tryptophan metabolism | D12 |
| Tryptophan catabolic process | GO:0006569 | 0.0072 | Tryptophan metabolism | D12 |
| Pancreatic secretion, secretory enzymes | GO:0019318 | 0.0273 | Carbohydrate metabolism | D13 |
| Alpha-amylase activity | GO:0035860 | 4.48E-05 | Carbohydrate metabolism | D13 |
| Starch and sucrose metabolism | GO:0007411 | 0.0011 | Carbohydrate metabolism | D13 |
| Carbohydrate digestion | GO:0000165 | 0.0015 | Carbohydrate metabolism | D13 |
| Salivary secretion | GO:0048483 | 0.0018 | Carbohydrate metabolism | D13 |

**Supplementary Table 3.** Complete list of enriched down-regulated GO terms and their associated GO number, summary group, and cluster, organized from smallest to largest FDR. Color coding correlates with summary group coloring as seen in the cluster figures within the primary manuscript.
