# Supplementary figures and images for: The proteomic fingerprint in infants with single ventricle heart disease in the interstage period: evidence of chronic inflammation and widespread activation of biological networks
Source: Front Pediatr. 2023 Dec 8;11:1308700. doi: 10.3389/fped.2023.1308700 (PMC10748388; doi:10.3389/fped.2023.1308700)

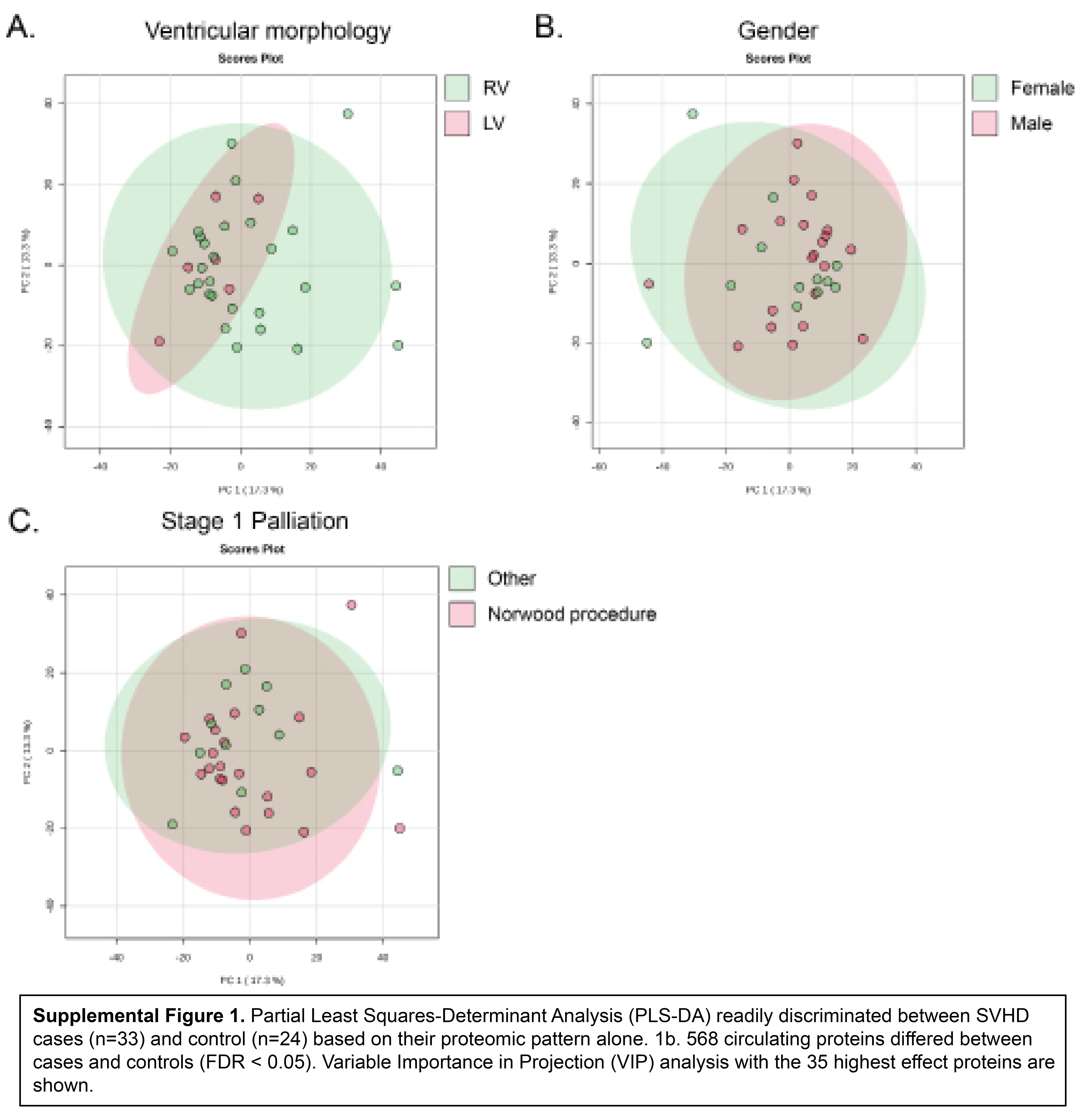

Supplement: Supplementary file 1 [file Datasheet1.zip › Datasheet1/Supplementary Files/Supplementary Figure 1.jpg]
